# How to make sense of 3D representations for plant phenotyping: a compendium of processing and analysis techniques

**DOI:** 10.1186/s13007-023-01031-z

**Published:** 2023-06-23

**Authors:** Negin Harandi, Breght Vandenberghe, Joris Vankerschaver, Stephen Depuydt, Arnout Van Messem

**Affiliations:** 1 Center for Biosystems and Biotech Data Science, Ghent University Global Campus, 119 Songdomunhwa-ro, Yeonsu-gu, Incheon, South Korea; 2 Department of Applied Mathematics, Computer Science and Statistics, Ghent University, Krijgslaan 281, S9, Ghent, Belgium; 3 BASF Innovation Center, Technologiepark 101, Zwijnaarde, Belgium; 4 Erasmus Applied University of Sciences and Arts, Campus Kaai, Nijverheidskaai 170, Anderlecht, Belgium; 5 Department of Mathematics, Université de Liège, Allée de la Découverte 12, Liège, Belgium

**Keywords:** Plant phenotyping, 3D acquisition, Computer vision, Point cloud processing, Segmentation, Skeletonization

## Abstract

Computer vision technology is moving more and more towards a three-dimensional approach, and plant phenotyping is following this trend. However, despite its potential, the complexity of the analysis of 3D representations has been the main bottleneck hindering the wider deployment of 3D plant phenotyping. In this review we provide an overview of typical steps for the processing and analysis of 3D representations of plants, to offer potential users of 3D phenotyping a first gateway into its application, and to stimulate its further development. We focus on plant phenotyping applications where the goal is to measure characteristics of single plants or crop canopies on a small scale in research settings, as opposed to large scale crop monitoring in the field.

## Introduction

Plant phenotyping, the quantitative measurement and assessment of plant features, is at the forefront of plant research, plant breeding, and crop management. In recent years, the use of non-destructive, image-based plant phenotyping methods has emerged as an active area of research, driven by improvements in hardware as well as software. Indeed, the emergence in the consumer market of low-cost, powerful image acquisition devices have made (raw) phenotyping data readily available and computational breakthroughs such as deep learning [1, 2] have in turn allowed researchers and plant breeders to readily obtain quantitative insights from data. Combined together, these improvements in computational plant phenotyping have reduced the reliance on tedious, manual intervention in data acquisition and processing and have enabled the use of automation in the laboratory and in the field.

One noteworthy development is the adoption of three-dimensional (3D) plant phenotyping methods [3]. Advancements in 3D image acquisition and processing methods are increasingly being applied and explored in the agricultural industry: automation and robotics are entering agriculture. Examples are autonomous and targeted harvesting, weeding, and spraying [4]. In agricultural biotechnology, there is a continuing effort to efficiently modify or select for traits like increased yield, drought tolerance, pest resistance and herbicide resistance, by linking the genotype with the phenotype [4, 5]. In precision farming, crop management is being optimized and made more flexible through monitoring and mapping of crop health indicators and environmental conditions [6, 7]. All these advancements require powerful vision systems, and applications in the different domains of phenotyping, inspection, process control, or robot guidance benefit from a 3D approach over 2D.

Compared to two-dimensional methods, 3D reconstruction models are more data-intensive but give rise to more accurate results. They allow for the geometry of the plant to be reconstructed [8], and hence find important applications in the morphological classification of plants. Moreover, 3D methods are also better able to track plant movement, growth, and yield over time [8–10], something that is hard to do with 2D approaches alone. These 3D reconstructed plant models could be used to, for example, describe leaf features, discriminate between weed and crop, estimate the biomass of the plant, and classify fruits [11]. In some cases, 3D methods that incorporate data from multiple viewing angles may provide insights that are hard or impossible to get from a 2D model alone, such as resolving occlusions and crossings of plant structures by reconstructing the plant distance, orientation, and illumination [2, 12–14].

These 3D reconstruction models can be classified in several ways. One such classification makes the distinction between rigid and non-rigid reconstruction. In rigid 3D reconstruction, the objects in the scene are static, while in non-rigid 3D reconstruction, the objects are dynamic and the method allows for some level of movement. Another possible classification, which is typical for agriculture (and thus also applicable in our case), will make the distinction between 3D reconstruction models for (controlled) indoor environments and outdoor environments that make use of images from the field [15].

**Fig. 1:**
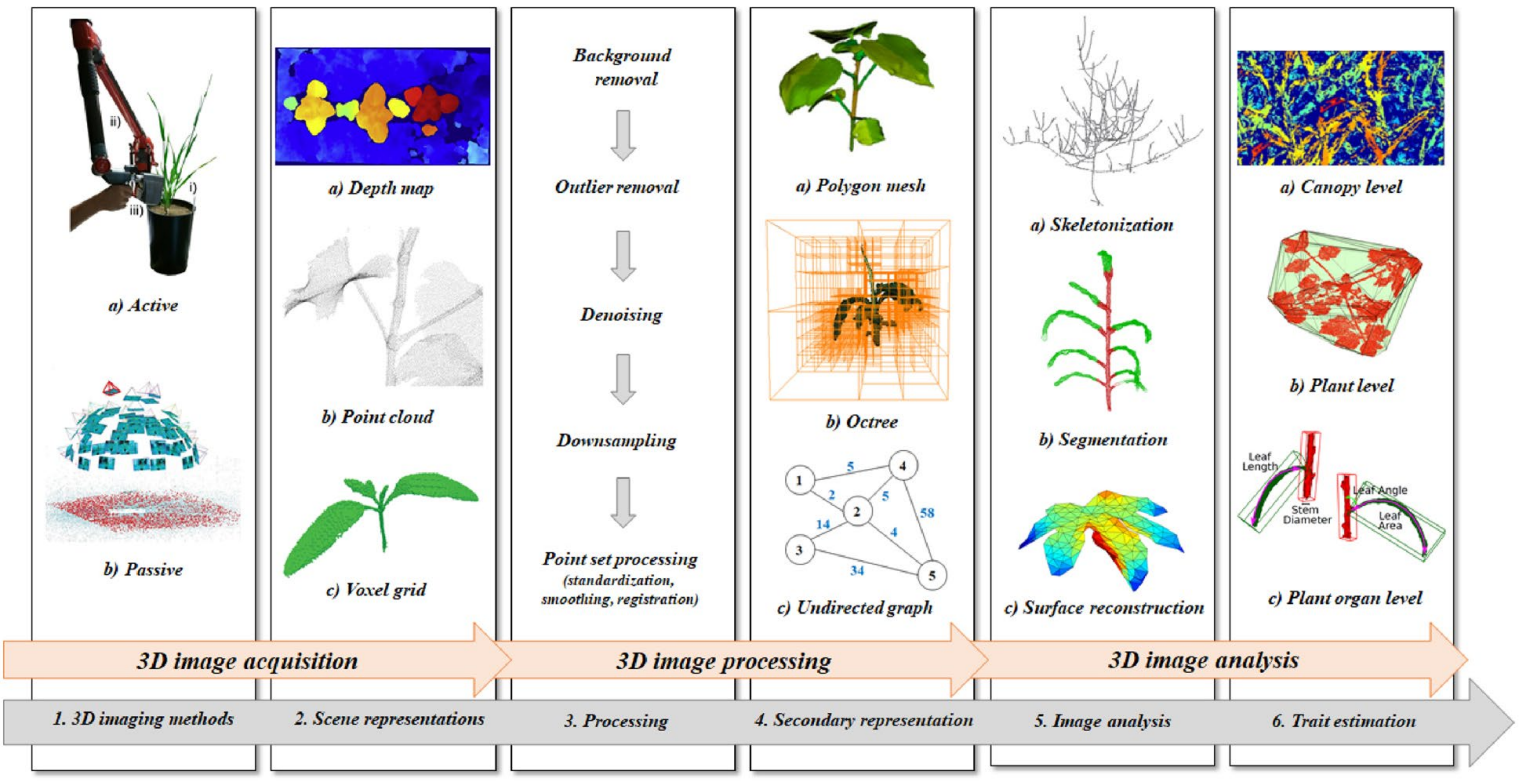
Schematic outline of typical processing and analysis steps for 3D plant phenotyping. Through an active (1.**a**) or passive (1.**b**) 3D acquisition method, either a depth map (2.**a**), a point cloud (2.**b** ) or a voxel grid (2.**c**) is obtained. After a number of preprocessing steps consisting of background subtraction, outlier removal, denoising and/or downsampling, the primary 3D representation may be transformed into a secondary representation, such as a polygon mesh (4.**a**), an octree (4.**b**), or an undirected graph (4.**c**), which facilitates further analysis. The main analysis steps, which may consist of skeletonization (5.**a**) segmentation (5.**b**) and/or surface fitting (5.**c**), precede measurements on the canopy (6.**a**), plant (6.**b**), or plant organ (6.**c**) level. 1.**a** [30], 1.**b** [169], 2.**b** [35], 2.**c** [101], 4.**a** [164], 4.**b** [104], 4.**c**, 6.**a** [108], and 6.**b** [85] reprinted under the terms of the Creative Commons Attribution 4.0 International License (http://creativecommons.org/licenses/by/4.0). 2.**a**, reprinted from [79], ©2015, with permission from Elsevier. 5.**a** [179] reprinted with permission from the American Society for Photogrammetry and Remote Sensing, Bethesda, Maryland (https://www.asprs.org/). 5.**b**, ©2017 IEEE, reprinted with permission from [227]. 5.**c** [348] and 6.**c** [248] reprinted with permission from the author

The set of problems that may arise during the processing and analysis of 3D representations, in general, is very large. For the analysis of 3D representations of plants in particular, a diverse set of tools is required because of the complexity and the non-solid characteristics of plant architecture, and its diversity both across and within species. It is our goal to point out typical processing and analysis steps, and to review methods which have been applied before, or could typically be used, in each of these steps. We will focus on applications for plant phenotyping where the ultimate goal is to measure phenotypic characteristics of single plants, or crop canopies on a small scale, as opposed to large scale yield and growth monitoring of crops in the field. We will not discuss the construction of virtual plant models where obtaining accurate or realistic 3D representations is a goal by itself. Nevertheless, many of the techniques used in that area can be applied for phenotyping as well. An outline of the topics covered in the present review is presented in Fig. 1.

## 3D image acquisition

**Fig. 2:**
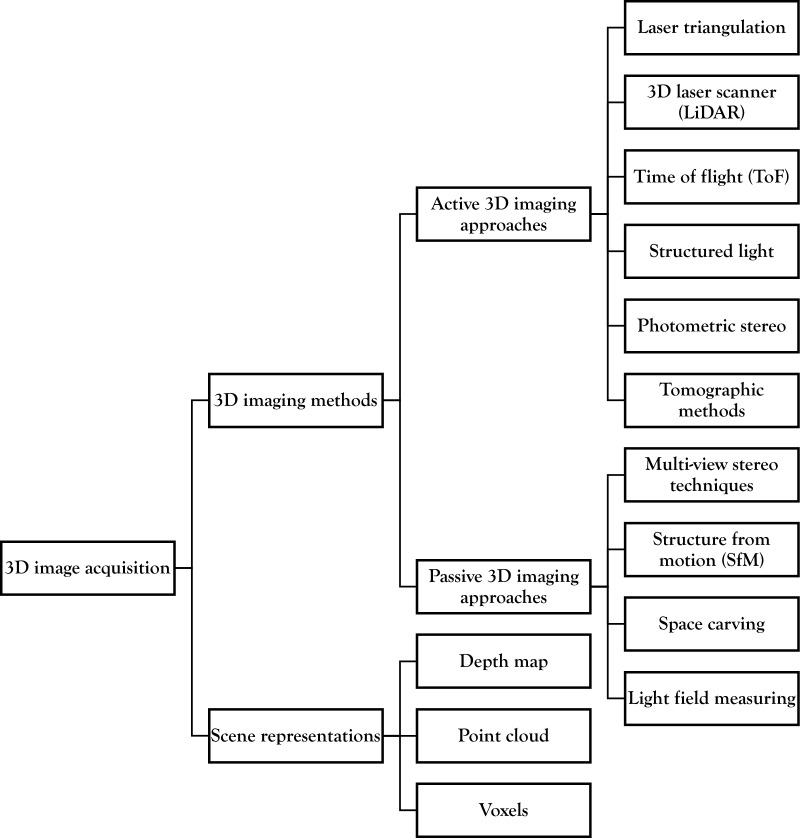
Overview of 3D image acquisition techniques

An overview of the topics covered in this section is presented in Fig. 2.

### 3D imaging methods

3D imaging methods can be classified roughly into active and passive approaches [16–23]. The active group refers to the techniques that use a controlled source of structured energy emissions, such as a scanning laser source or a projected pattern of light, and a detector like a camera. On the other hand, the passive techniques rely on ambient light in order to form an image [24]. Compared to 2D imaging, both passive and active 3D imaging approaches can significantly improve the accuracy of plant growth measurements and even expand on the architectural traits available. However, 3D imaging techniques still lack in several crucial areas such as speed, availability, portability, spatial resolution, and cost [3].

**Fig. 3:**
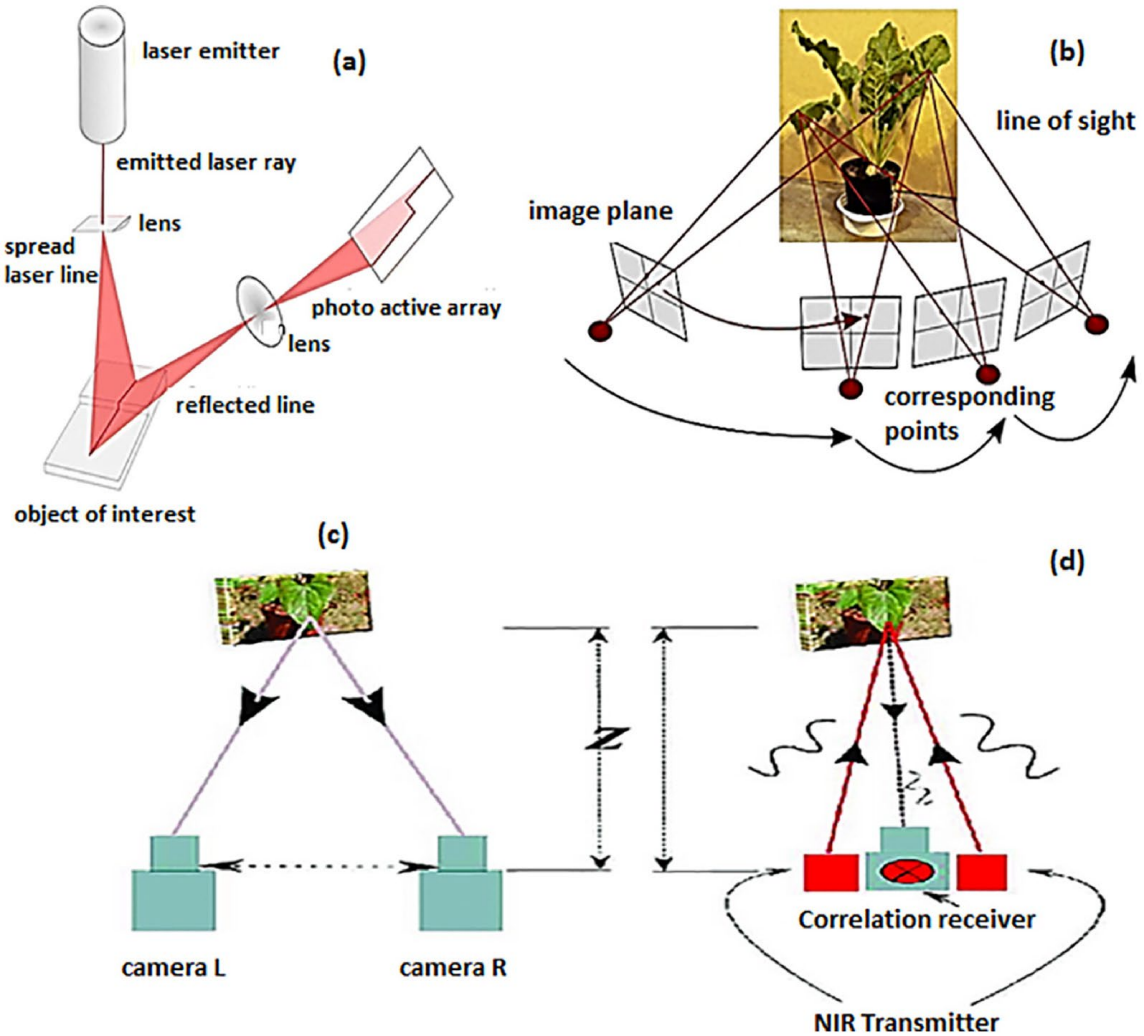
3D imaging system setup: **a** Laser triangulation, **b** Structure from Motion (SfM), **c** Stereo vision, and **d** Time-of-Flight (ToF). Reprinted from [8] under the terms of the Creative Commons Attribution 4.0 International License (http://creativecommons.org/licenses/by/4.0)

Typically, active 3D imaging methods require specialized measuring devices such as LiDAR, MRI or PET scanners, which are costly to acquire and maintain but result in highly accurate data. Passive imaging methods, on the other hand, tend to be more cost-effective as they typically use commodity or off-the-shelf hardware, but may result in comparatively lower-quality data that often require significant computational processing to be useful. The specific trade-offs between active and passive 3D imaging methods, in terms of cost and fitness for a specific purpose, are discussed in this section. A comparison of active and passive methods, and of imaging techniques covered in this paper is presented in Tables 1 and 2, respectively. A full list of papers and plants using these techniques can be found in Table 3, under the header “3D Image Acquisition and Registration”. Four selected techniques from these two categories are illustrated in Fig. 3.

**Table 1:** A comparison of 3D imaging methods

Type	Sensor	Principle	Advantages	Disadvantages	Output
Active	Uses active sensors such as structured light and laser scanners	Uses radiometric interaction with the object to directly capture a 3D point cloud	(1) Overcomes correspondence problems (2) High accuracy	(1) Limited to specific environments and illumination conditions (2) Requires specialized, expensive equipment (3) Lack of color information	(1) 3D point clouds (2) Depth maps
Passive	Uses passive sensors such as standard imaging cameras	Analyzes multiple images from different perspectives to generate a 3D point cloud	(1) Low cost (2) Includes color information	(1) Correspondence problems (2) Low accuracy (outliers and noise) (3) Computationally complex (4) Relatively slow	(1) 3D point clouds (2) Depth maps (3) Voxels

**Table 2:** A comparison of 3D imaging techniques

Method	Principle	Advantages	Disadvantages	Environment
Active methods				
3D Laser Scanner (LiDAR)	Determines the range by targeting an object or a surface using a laser to measure the time for the reflected light to return to the receiver	(1) High resolution (2) Low-cost devices available (3) Relatively insensitive to lighting conditions (4) Suitable for high volume scanning	(1) Complex scanning process (2) Requires calibration (3) Lack of depth detection mechanism	(1) Fields (2) Controlled environments
Laser Triangulation	Captures 3D data by pairing the laser light source with a sensor array (e.g., camera)	(1) High accuracy at close range (2) High resolution (3) Low cost (4) Relatively insensitive to the lighting conditions or surface textures	(1) Low accuracy at large distances (2) Computationally intensive (3) No color information (4) Difficult to scan transparent or reflective surfaces	(1) Fields (2) Controlled environments
Photometric Stereo (PS)	Estimates local surface orientation by using a sequence of images of the same surface from the same viewpoint but under illumination from different directions	(1) High resolution (2) Low cost (3) High speed (4) Reasonable computational cost	(1) Difficult to handle shiny and semi-translucent surfaces (2) Requires calibration	(1) Fields (2) Controlled environments
Structured Light	Projects a series of known patterns onto the object and builds up a 3D image by measuring the deformation of the patterns	(1) High accuracy (2) High-speed scanning (3) High resolution	(1) Vulnerable to ambient light interference (2) Decreasing accuracy as the measurement distance increases	(1) Controlled environments
Time of Flight (ToF)	Builds up a 3D image using light emitted by a laser or LED source and measuring the roundtrip time between the emission of a light pulse and the reflection	(1) Easy setup due to the small size of the camera (2) High-speed measurement (3) Wide measurement range (4) Relatively insensitive to ambient light	(1) Expensive (2) Difficulties with shiny surfaces	(1) Fields (2) Controlled environments
Tomographic Methods	Create a series of 2D slices in order to generate a 3D volume	(1) High resolution (2) High accuracy	(1) Not suitable for large-scale field studies (2) Time-consuming (3) High cost	(1) Controlled environments
Passive methods				
Light Field Measuring	Reconstructing the 3D information of a scene by capturing both the radiant intensity and the direction of the incoming light in one plane	(1) High-dimensional representation	(1) Technical complexity (2) Computational cost	(1) Fields (2) Controlled environments
Multi-view Stereo (MVS) Techniques	Uses two or more cameras to generate parallax from different perspectives to obtain distance information about an object	(1) Simplicity of use (2) Low cost (3) High accuracy	(1) Limited imaging range (2) Low imaging quality (3) Computationally intensive (4) Poor performance in real-time (5) Relatively sensitive to the environment conditions	(1) Fields (2) Controlled environments
Space Carving	Uses a voxel grid and information from pictures taken from different perspectives to remove voxels which are not part of the object	(1)Avoids correspondence problem (2) Low cost (2) Requires fewer images than, e.g., SfM to obtain a good representation	(1) Calibration requirements (2) Quality of reconstruction depends on the number of views (3) Exact segmentation requirements (4) Lack of depth detection mechanism (5) Not suitable for highly non-convex objects	(1) Controlled environments
Structure from Motion (SfM)	Captures 3D information from sequences of overlapping 2D images	(1) Low cost (2) High resolution (3) High color reproduction (4) Wide measurement range (5) Mature algorithm, frequently used (6) Some degree of automatic calibration	(1) Need to move measurement equipment continuously (2) Reconstruction complexity (3) Time-consuming	(1) Fields (2) Controlled environments

**Table 3:** Well-established methods and algorithms used for 3D plant phenotyping

Method	Plant	Short description
**3D Image Acquisition and Registration**		
3D Laser Scanning (LiDAR)	Aubergine [252] Bamboo-leaf oak tree [252] Barley [30, 156] Benth (Nicotiana benthamiana) [183] Birch [174] Botanic trees [168] Cereal plants [27] Chickpea [328] Elm tree [182] Grape [329] Grapevine [35] Horse Chestnut [174] Japanese cedar [252] Japanese larch [252] Maize (Corn) [34, 129, 160, 186, 330, 331] Orchard tree [175] Poplar [174] Rapeseed (Brassica sp.) [32] Red Oak [174] Rosebush [264] Sorghum [328] Soybean [25] Sugar beet [25, 36] Sugar maple [182] Sweet Chestnut [174] Thale cress (Arabidopsis) [10, 28, 156] Tomato [34, 160, 183] Wheat [31, 35, 36] Yellow birch [182] Others [205, 332]	Measures accurately the distance between the sensor and a target based on the elapsed time between the emission and return of laser pulses (’Time-of-Flight’ (ToF) method) or based on trigonometry (the ’optical probe’ or ’light section’ methods).
Electrical Resistance Tomography (ERT)	Chicory [56] Maize (Corn) [56] Switchgrass [56]	Is a geophysical technique for imaging sub-surface structures from electrical resistivity measurements made at the surface, or by electrodes in one or more boreholes.
Gaussian Mixture Model (GMM)	Apple tree [185] Barley [121] Cherry [185] Thale cress (Arabidopsis) [185]	Represents discrete point sets by continuous density functions [157].
Generalized Voxel Coloring		Variant of Voxel Coloring which allows the cameras to completely surround the scene [119].
Iterative Closest Point (ICP)	Barley [156] Maize (Corn) [34, 38, 47, 129, 227] Pepper [155] Rapeseed (Rape) [33, 125] Thale cress (Arabidopsis) [156] Tomato [34]	Minimizes distances between two point clouds. Often used to obtain a full 3D reconstruction from multiple 3D scans which capture the object from different angles [112, 146–149].
Magnetic Resonance Imaging (MRI)	Barley [63] Bean plant [62] Maize (Corn) [63]	Is a type of scan that uses strong magnetic fields and radio waves to produce detailed images of the inside of the body.
Marching Cubes		Transforms a voxel grid into a polygon mesh. The algorithm walks through the voxel grid and chooses a certain surface configuration composed of triangles from a table, based on the values of neighboring voxels. The individual polygons are then fused into a surface [162].
Multi-view Stereo (MVS)	Anthurium andraeanum [40] Barley [103, 108] Banana [90] Basil [317] Calathea makoyana [111] Epipremnum aureum [111] Grapevine [333] Hedera nepalensis [111] Ipoestia [317] Ixora [317] Litchi [88] Maize (Corn) [107, 133, 227, 330, 334] Monstera deliciosa [111] Rape [89] Soybean [77, 170, 334] Sugar beet [108] Tomato [2, 85, 101] Wheat [169]	Uses two or more cameras to generate parallax from different perspectives, and obtaining the distance information of the object and then reconstructs a 3D shape from calibrated overlapping images captured from different viewpoints [170]. In case of having two cameras, it is called stereo vision.
Patch-based Multi-View Stereo (PMVS)	Artificial plant [75] Bambara groundnut [78] Proso millet [78] Rice [75, 76] Wheat [75, 76]	Reconstructs a 3D point cloud model based on multiple color input images. A requirement of this algorithm is that the intrinsic (such as focal length) and extrinsic (3D position and orientation) camera parameters are known.
Photometric Stereo (PS)	Dock (Rumex Obtusifolius) [12] Potato [12] Thale cress (Arabidopsis) [17]	Estimates the surface normals of objects by observing the object under different lighting conditions [50].
Shape-from-silhouette (SFS) *(Shape-from-contour)*	Artificial plant [209] Barley [121] Maize (Corn) [121, 123] Sorghum [209] Tomato [101] Wheat [209]	Reconstructs the visual hull of an object, by means of the intersection of silhouette cones determined by the objects’ silhouettes captured from different angles [335].
Space Carving *(Shape-from-contour)*	Aloe vera [81] Banana [104] Bromeliad species [81] Chili [81] Cordyline species. [81] Cotton [99] Maize (Corn) [99, 104] Pumpkin (Cucurbita pepo) [81] Rapeseed [81] Sorghum [105] Other [102]	Reconstructs the maximal shape of an object that is photo-consistent with the object. Photo-consistency includes consistency with the objects silhouettes, but also with its projected surface colors. The algorithm iteratively ’carves’ space away from an enclosing volume in a well-defined way, until the shape is photo-consistent with all the views [98].
Structured Light	Anthurium [48] Cabbage [26] Cucumber [26] Dishlia [48] Tomato [26]	Extracts the 3D surface shape based on the information from the distortion of the projected structured-light pattern without ionizing radiation [52].
Structure from Motion (SfM)	Barley [18] Basil [317] Brussels sprout [79] Chili plant [11] Grapevine [221, 333] Ipoestia [317] Ixora [317] Maize (Corn) [9, 13, 82, 334, 336–338] Nephthytis [94] Olive [223] Physalis sp. [9] Poinsettia [94] Brassica sp. (Rapeseed) [9] Savoy cabbage [79] Schefflera [94] Soybean [14, 83, 84, 84, 334, 339] Sugar beet [13] Sunflower [11, 13, 79, 83, 240] Thale cress (Arabidopsis) [9] Tomato [11, 85, 338] Wheat [9] Other [135, 340]	Reconstructs the 3D structure using a series of 2D images with a high degree of overlap, taken from different angles. It identifies matching features which are tracked from image to image to produce estimates of the camera positions and orientations, as well as the coordinates of the features to create a point cloud.
Time of Flight (ToF) (Including Microsoft Kinect sensors)	Apple tree/orchard [110, 341] Calathea makoyana [111] Cyclamen [40] Epipremnum aureum [111] Hedera nepalensis [111] Hydrangea [40] Lettuce [44] Maize (Corn) [13, 38, 47] Monstera deliciosa [111] Orchidaceae [40] Paprika [109] Pelargonium [40] Pepper [155] Pumpkin [46] Rapeseed [33] Rosebush [110] Sorghum [39] Soybean [43] Sugar beet [13, 36] Sunflower [13] Tomato [45] Wheat [36] Yucca [110]	ToF cameras use time between emitted light and reflected light from thousands of points to conduct 3D images.
Voxel Coloring *(Shape-from-photoconsistency)*	Rose [97]	Reconstructs a photo-consistent 3D shape not by carving away voxels, but by identifying voxels that have a unique coloring which is constant across all possible photo-consistent interpretations of the scene. Has the limitation that all cameras have to be placed on one side of the scene.
X-Ray (Micro) Computed Tomography (CT / µCT)	Barley [64] Bean plant [59, 62] Cassava [59] Chickpea [64] Duckweed [57] Maize (Corn) [58, 61, 337] Sinningia [342] Sorghum [343] Tomato [61] Wheat [60, 61, 64] Other [344]	Is a non-destructive imaging tool for the production of high-resolution three-dimensional (3D) images composed of two-dimensional (2D) trans-axial projections, or ‘slices’, of a target specimen [345].
**3D Image Processing**		
Bilateral smoothing techniques	Maize (Corn) [15] Tomato [346]	Is a non-linear filtering technique and a simple, non-iterative scheme for edge-preserving smoothing [137, 142].
Clustering-based Segmentation	Grapevine [35, 221] Olive [223] Wheat [35]	Uses clustering algorithms to group data points that are more similar to one another in order to obtain a segmented image.
Color-based Filter	Brassica sp. [9] Calathea makoyana [111] Epipremnum aureum [111] Hedera nepalensis [111] Maize (Corn) [9] Monstera deliciosa [111] Physalis sp. [9] Thale cress (Arabidopsis) [9] Wheat [9]	Distinguishes between foreground and background and removes background pixels based on the RGB color information.
Dart Throwing Filter		Sequentially add points from the original point cloud to a downsampled point cloud if they don’t have a neighbor in the output point cloud within a certain radius [136].
Density-based Spatial Clustering of Applications with Noise (DBSCAN)	Maize (Corn) [34, 82, 161] Tomato [34, 161]	Removes clusters of size less than a predetermined threshold if they are located further away than a certain distance from any other point cluster. Can be used as noise filtering.
Mean Shift	Paprika [109]	Iteratively shifts each data point to the average of data points in its neighborhood by using kernel density estimation.
Moving Least Squares (MLS)	Pepper [155]	Iteratively projects points on weighted least squares fits of their neighborhoods to cause the points to lie closer to an underlying surface [131].
M-Estimator Sample Consensus (MSAC)	Maize (Corn) [130]	Is a variant of the RANSAC algorithm which adopts bounded loss of RANSAC by using a different loss function [347]
Radius-based Outlier Filter (RBOF)	Calathea makoyana [111] Epipremnum aureum [111] Hedera nepalensis [111] Monstera deliciosa [111]	Modifies the elemental criterion of a specific element based on a weighted average of the criteria in a fixed neighborhood.
Random Sample Consensus (RANSAC)	Grape [329] Maize (Corn) [82] Soybean [77, 170] Other [135, 205]	Fits geometric primitives to point clouds by choosing the best among fits to numerous random samplings of the data [128].
Spatial Region Filter	Calathea makoyana [111] Epipremnum aureum [111] Hedera nepalensis [111] Maize (Corn) [133] Monstera deliciosa [111]	Removes all points outside a region defined in a 3D coordinate system.
Statistical Outlier Removal (SOR) Filter	Calathea makoyana [111] Epipremnum aureum [111] Hedera nepalensis [111] Maize (Corn) [15, 38] Monstera deliciosa [111] Soybean [25, 84] Sugar beet [25] Other [135]	Removes points if the mean distance to its neighbors surpasses a threshold based on the mean and standard deviation of all neighbor distances.
Surface Boundary Filter (SBF)	Calathea makoyana [111] Epipremnum aureum [111] Hedera nepalensis [111] Monstera deliciosa [111]	Identifies and removes boundary points using a threshold on the angle between a projected vector in the normal plane to the first two principal components and one of the principal components [111].
Voxel Grid Downsampling	Brassica sp. [9] Calathea makoyana [111] Epipremnum aureum [111] Hedera nepalensis [111] Maize (Corn) [9, 130] Monstera deliciosa [111] Physalis sp. [9] Thale cress (Arabidopsis) [9] Wheat [9]	Divides the point cloud into a 3D voxel grid and replaces points within each voxel by the centroid of all the points within the voxel [134].
**3D Image Analysis**		
α-shape Triangulation	Thale cress (Arabidopsis) [156] Barley [156]	Transforms a point cloud into a polygon mesh. The shape is determined by connecting sets of 3 points into a triangle if they can be circumscribed by a sphere with radius α which doesn’t contain any other points [163].
Breath-first flood-fill algorithm	Tomato [101]	Determines the area connected to a given node in a multi-dimensional array.
Constrained Region-growing	Cotton [164]	Segments a surface mesh segmentation by growing regions from seed points to adjacent mesh faces, constrained by changes in curvature, which correspond to sharp edges [207, 208].
Delaunay Triangulation *(Advancing Front)*	Aloe vera [81] Brassica sp. [81] Bromeliad sp. [81] Chili [81] Cordyline sp. [81] Maize (Corn) [15, 229] Pumpkin (Cucurbita pepo) [81] Rape [81] Rice [229]	Creates a triangulation (in 2D) or covering by tetrahedra (in 3D) of a point cloud, such that no point lies in the circumcircle of any triangle or tetrahedron. Delaunay triangulations tend to maximize the minimum interior angle of each triangle or tetrahedron, and hence avoid sharp angles (“sliver triangles”). Used in the context of this paper to grow a surface from a set of seed triangles. [233–235].
Dense Conditional Random Field (CRF)	Maize (Corn) [227]	Acquires an accurate and spatially consistent labeling of pixels after the application of a unary classifier which doesn’t take the spatial context of pixel labels into account. The model establishes pairwise potentials on all pairs of pixels in the image. An energy function consisting of both unary and pairwise components is minimized [228].
Dijkstra’s algorithm	Berryless grape [247] Maize (Corn) [130] Pine tree [180]	Finds the shortest path between vertices in a graph [171].
(Fast) Point Feature Histogram (FPFH / PFH)	Barley [226] Benth (Nicotiana benthamiana) [183] Grapevine [226] Maize (Corn) [34, 161, 227] Rapeseed (Rape) [125] Tomato [34, 161, 183] Wheat [226] Other [224, 225, 340]	Describes the local geometry around a point in point clouds using features based on the angular relationships between pairs of points and their normals, within a certain radius around each query point. The features are counts within histogram bins of these values. FPFH is a more efficient version of PFH which reduces the number of pairs for which angles have to be calculated while retaining most of the discriminative power of PFH.
Locally Estimated Scatterplot Smoothing (LOESS)	Maize (Corn) [229] Rice [229]	Reconstructs a continuous surface even with the presence of the discontinuity of surface points.
Minimum Oriented Bounding Box (MOBB)	Rapeseed (Rape) [125]	Determines the smallest bounding box for a point set (i.e., smallest area, volume or hyper-volume in higher dimensions) within which all points lie.
Minimum Spanning Tree		Finds a subset of edges in a graph which connects all the vertices, and which has a minimum total length [172].
Non-Uniform Rational B-splines (NURBS)	Mint [238] Maize (Corn) [38] Sunflower [239] Other [237]	Defines smooth curves and surfaces by a list of 3D coordinates of surface control points and associated weights .
Randomly Intercepted Nodes (RAIN)	Maize (Corn) [82]	Simulates the behavior of randomly placed rain drops to find the routes of these drops while moving from point to point. Performs segmentation based on the points considered as potential path candidates.
Spectral Clustering	Thale cress (Arabidopsis) [9] Birch [174] Brassica sp. [9] Horse Chestnut [174] Maize (Corn) [9] Physalis sp. [9] Poplar [174] Red Oak [174] Sweet Chestnut [174] Wheat [9]	Is a technique with roots in graph theory, where the approach is used to identify communities of nodes in a graph based on the edges connecting them.
Voxel verlapping Consistency Check	Cotton [99] Maize (Corn) [99]	Encloses the voxel grid by a bounding box. Considers the (area of) constituent voxels at different cross-sections of this bounding box to segment between stem and leaves.
**Machine Learning Techniques**		
Boosting	Soybean [84]	Seeks to improve the prediction power by training a sequence of weak models, each compensating the weaknesses of its predecessors.
Deep Learning (DL)	Banana [90] Maize (Corn) [160, 299, 310, 313] Rice [311] Rosebush [264] Rosette plants [1] Sorghum [276] Thale cress (Arabidopsis) [17] Tobacco [276] Tomato [2, 160, 276, 301]	DL is a very commonly employed algorithm in the ML algorithms, and it is derived from the conventional neural network but considerably outperforms its predecessors. DL employs transformations and graph technologies simultaneously in order to build up multi-layer learning models. The most famous types of deep learning networks are CNNs, RNNs, and RvNNs.
Hidden Markov Models (HMMs)	Maize (Corn) [34] Tomato [34]	Are probabilistic models in which an unobservable (“hidden”) Markov process influences an observable process in a specific way. The goal is to est the hidden states from the observations.
K-Means Clustering	Barley [226] Grapevine [226] Maize (Corn) [117] Sorghum [117] Soybean [84] Wheat [226]	Is one of the simplest and most popular unsupervised machine learning algorithms and aims to partition *n* observations into *k* clusters in which each observation belongs to the cluster with the nearest mean, serving as a prototype of the cluster.
K-Nearest Neighbors (KNN)	Aloe vera [81] Birch [174] Brassica sp. [81] Bromeliad species [81] Chili [81] Cordyline sp. [81] Horse Chestnut [174] Maize (Corn) [34, 133, 161, 186] Poplar [174] Pumpkin [81] Rapeseed [81] Red Oak [174] Sweet Chestnut [174] Tomato [34, 161]	Is a simple, supervised machine learning algorithm that can be used to solve both classification and regression problems and clusters the point set into a series of *k* nearest neighbors.
Random Forest Classifier (RFC)	Rosebush [264] Other [135, 344]	Uses a combination of tree predictors such that each tree depends on the values of a random vector sampled independently and with the same distribution for all trees in the forest [263].
Self-Organizing Map (SOM)	Maize (Corn) [34, 161] Tomato [34, 161]	Is an unsupervised neural network using the concept of competitive learning instead of back-propagation.
Support Vector Machine (SVM)	Maize (Corn) [34, 161, 227] Soybean [84] Tomato [34, 161]	Is a popular and commonly used choice for binary classification problems which can perform nonlinear classification.
**Miscellaneous Techniques**		
	Maize (Corn) [95] Mango [122] Olive [122] Peach [122] Pine tree [180] Tomato [242] Walnut [122]	Methods developed for specific plant applications.

#### Active 3D imaging approaches

Active approaches use active sensors [25] and rely on radiometric interaction with the object by, e.g., using structured light or laser [23] to directly capture a 3D point cloud that represents the coordinates of each part of the subject in the 3D space [25]. Triangulation, Time of Flight (ToF, discussed below), and phase-shift are all examples of active measurement techniques [18]. Structured light [26] and laser scanners [10, 27, 28] are active technologies that are based on triangulation to determine the point locations in a 3D space [17]. Because active 3D imaging approaches rely on emitted energy, they can overcome several problems related to passive approaches such as correspondence problems (i.e., the problem of ascertaining which parts of one image correspond to which parts of another image, where differences are due to movement of the camera, the progress of time, and/or movement of objects in the photos). Furthermore, active 3D acquisition techniques can provide higher accuracy, but they require specialized and often expensive equipment. Because of their reliance on a radiation source, the environment and the illumination conditions in which active techniques can be used are often limited.

Other possible drawbacks are that approaches using structured light require very accurate correspondence between images while laser scanners can be slow and can potentially heat or even damage plants at high frequencies.

*Laser triangulation* These techniques involve shining a laser beam to illuminate the object of interest and a sensor array to capture laser reflection [8]. Due to the low-cost setup, they are widely used in laboratory experiments [29, 30]. Paulus et al. [30] used this technique to produce a 3D point cloud of barley plants. Likewise, Virlet et al. [31] used this technique for producing point clouds from wheat canopies and Kjaer and Ottosen [32] for rapeseed.

*3D laser scanner (LiDAR)* A 3D laser scanner is a high-precision point cloud acquisition instrument. However, the scanning process is complex and requires calibration objects or repeated scanning to accomplish the point cloud registration and stitching [33]. Chebrolu et al. [34] used a laser scanner to record time-series data of tomato and maize plants over a period of two weeks, while Paulus et al. [35] used a 3D laser scanner to create point clouds of grapevine and wheat.

Low-cost laser scanning devices, such as the Microsoft Kinect sensor and the HP 3D Scan system, are readily available on the consumer market and have been widely used for plant characterization in agriculture [13]. Although these provide lower resolutions, they may still be sufficient for less demanding applications [36], and they are designed for use in a wide range of ambient light conditions.

Terrestrial laser scanners (TLS) allow for large volumes of plants to be measured with relatively high accuracy, and are therefore mostly used for determining parameters of plant canopies and fields of plants. However, acquiring and processing TLS data is time consuming and costly due to the large data volumes involved [8, 33, 37].

*Time of flight (ToF)* ToF cameras use light emitted by a laser or LED source and measure the roundtrip time between the emission of a light pulse and the reflection from thousands of points to build up a 3D image [8]. Examples of this method can be found in the works of Chaivivatrakul et al. [38] on maize plants, Baharav et al. [39] on sorghum plants, and Kazmi et al. [40] on a number of different plants including cyclamen, hydrangea, orchidaceae, and pelargonium.

Some ToF devices available on the consumer market, such as the Kinect [41] (through the KinectFusion algorithm [42]), provide a convenient and cost-effective way to perform 3D reconstruction in real time [43].

Using Kinect for acquiring a 3D point cloud data can be found in several studies including Wang et al. [44] on lettuce, González et al. [45] on tomato seedling, Zhang et al. [46] on pumpkin roots, and Zhang et al. [47] on maize plants.

All in all, using close range photogrammetry for a real-time follow-up produces highly detailed models, but it results in a higher processing time compared to the other methodologies. Increasing computational power would allow for rapid model processing that is able to analyze growth dynamics at higher resolutions in the case of photogrammetry [13].

*Structured light* Structured light cameras project a pattern, for example a grid or a specific pattern of horizontal bars, to capture 2D images and convert them into 3D information by measuring the deformation of the patterns [8]. Li et al. [48] used an acquisition system consisting of a standard structured light scanner to capture the geometry of the dishlia plant by looking at it from different angles. To obtain this result, they used a turntable to rotate the plant by 30 degrees at a time. A complete review of using structured light methods for high-speed 3D shape measurement can be found in [49].

*Photometric stereo (PS)* Pioneered by Woodham [50], PS is a low-cost active imaging technique that can achieve high-resolution images and fast capture speeds. PS estimates local surface orientation by using a sequence of images of the same surface from the same viewpoint but under illumination from different directions. This technique uses data from several images and is therefore able to circumvent some of the problems that plague Shape-from-shading [51] approaches (not applied in plant phenotyping as far as we know) [52–55]. Bernotas et al. [17] used this technique for tracking the growth for the thale cress plant.

*Tomographic methods* These methods create a series of 2D slices to generate a 3D volume and provide non-destructive, high-resolution data of external and internal structures or even the movement of small molecules through a root system in the case of plants. X-ray computed tomography (CT), magnetic resonance imaging (MRI), and positron emission tomography fall into this category [56].

MRI and CT, which are usually applied in the medical imaging domain, can also be used to visualize plant root systems within their natural soil environment [56–65]. Applications of CT during the last 30 years show considerable effectiveness for the visualization of root structures. Fine root structures can be visualized using micro-computed tomography ($\mu \hbox {CT}$) devices, which offer high resolving powers, down to 50 $\mu$m [66].

These methods produce voxels which contain intensity information, either representing the capacity of the material to absorb and emit radio frequency energy in the presence of a magnetic field in case of MRI, or the capacity of the material to absorb the X-ray beam in case of CT.

Neutron tomography (NT) complements other techniques like CT or nuclear MRI, due to the specific attenuation characteristics of thermal or cold neutrons [67, 68]. As neutrons are attenuated by the presence of water, while passing through volumes of silicon-based material in a relatively unimpeded way, NT presents an attractive method for the phenotyping of plant roots embedded in soil, modeling the rhizosphere, and quantifying the spatial distribution of water in the soil-plant system with high precision and good spatial resolution.

For example, Krzyzaniak et al. [66] used NT to provide a 3D reconstruction of grapevine roots and sand in an aluminum sample holder, while Moradi et al. [69] used NT to study root developments in soil of different texture and showed that sandy soil was the best to obtain a good contrast of the root visualization. Compared to X-ray imaging, NT has advantages and disadvantages. Due to its ability to penetrate bulk volumes of soil and rubble, NT is able to visualize water dynamics [69–74]. However, NT is a more labor-intensive process that requires highly specialized equipment, and produces images of comparatively lower resolution.

#### Passive 3D imaging approaches

Passive methods use passive sensors such as cameras and rely on analyzing multiple images from different perspectives to generate a 3D point cloud [21, 22, 25]. They capture plant architectures without introducing new energy (e.g., light) into the environment. Multi-view stereo (MVS) [75, 76], of which the most common application is binocular stereo [77, 78], Structure from Motion (SfM) [79], light-field (plenoptic) cameras [80], and space carving [81] approaches are examples of methods and technologies using this approach [17]. Of these, SfM is widely in use, especially in the 3D reconstruction of plants [11, 13, 14, 18, 79, 82–85]. In this approach, multiple photographs are taken from different unknown angles after which the camera position and depth information are estimated simultaneously based on matched features in the images.

Compared to active techniques, these methods are cheaper and can be applied using standard imaging hardware, but they are prone to producing outliers and noise [86]. Another disadvantage is that they are computationally complex, and thus relatively slow. Because passive methods make use of ambient light reflections, they do gain color information in addition to 3D shape information, which is not readily available from active techniques unless when combined with another imaging system.

*Multi-view stereo techniques* These methods use two or more cameras to generate parallax from different perspectives and obtain distance information of the object through comparing these perspectives [87]. Although the structure of the binocular camera is simple, and the calculation speed is fast, the results are greatly affected by the environment and in particular the method struggles with scenes lacking texture information [88]. Xiong et al. [89] used binocular stereo cameras and a semi-automatic image analysis system to quantify the 3D structure of rape plants. Chen et al. [90] assembled two binocular vision systems into a four-camera vision system to construct a multi-view stereo system to perform multi-view 3D perception of banana central stocks in complex orchard environments. Rose et al. [85] utilized a multi-view stereo method to reconstruct tomato plants.

*Structure from motion (SfM)* This technique can estimate 3D models from sequences of overlapping 2D images and can automatically recover the camera parameters like focal length, distortion, position and orientation [91–94]. It has low-cost, high point cloud accuracy, and high color reproduction. However, it is cumbersome and time-consuming to shoot sequence images [33]. Using equipment available in most biology labs, such as cameras and turntables, Lou et al. [9] built an accurate multi-view image-based 3D reconstruction system that yields promising results on plants of different forms and sizes and applied it to different plants, including thale cress, Brassica sp., maize, Physalis sp., and wheat.

SfM is not limited to analyzing the plant stem and leaves. Liu et al. [95] developed an automatic 3D root phenotyping system consisting of a 3D root scanner and root analysis software for excavated root crowns of maize. Their system generates a model of the root system from a 3D point cloud and calculates 18 root-specific phenotypical traits from this model.

*Space carving* There exist different shape estimation methods [96], including voxel coloring [97] and space carving [98, 99]. Unfortunately, voxel coloring is guaranteed to work only if all of the cameras lie on the same side of the viewing plane, which precludes the use of more general configurations of cameras. To remedy this, Kutulakos and Seitz [98] generalized voxel coloring to space carving, an approach whereby a 3D scene is iteratively reconstructed by selecting subsets of photographs taken from the same side and removing voxels that are not consistent with the selected photographs [100]. The process ends when there are no more voxels to remove.

Some recent contributions focus on the phenotyping of seedlings [101–103] as they are easier to reconstruct, while others focus on accelerating voxel carving through the use of octrees [104]. Scharr et al. [104] then apply this accelerated method to maize and banana seedlings. Gaillard et al. [105] developed a high throughput voxel carving strategy to reconstruct 3D representations of sorghum from a small number of images.

In comparison to SfM, space carving requires fewer images and lower processing time. However, this method needs an exact calibration and segmentation of the object to reconstruct, whereas SfM can estimate calibration automatically. This method is therefore appropriate in a controlled environment, where an accurate calibration is attainable [105].

*Light field measuring* Compared to a standard camera, consisting of a main lens that focuses a scene directly onto an image plane, a lightfield camera generates an intermediate image which is focused on the image plane by a micro lens array. Light field cameras allow for images to be modified after recording, and therefore offer more flexibility in how an image is perceived. Polder et al. [106] used a lightfield camera to capture the depth map of tomato plants in a greenhouse. Apelt and Kragler [80] used a light field camera which provides two high-resolution grey-scale images (a focus image and a depth image containing metric distance information) to build a system in order to monitor spatio-temporal plant growth for thale cress.

### Scene representations

By choice, or depending on the acquisition method, 3D scenes and objects can be represented as a depth map, as a point cloud, or as a voxel grid.

#### Depth map

A depth map is a 2D image where the value of each pixel represents the distance from the camera or scanner (sometimes referred to as “2.5D”). In such representations, objects occluded by the projected surface are not measured. 3D image acquisition methods which may output depth maps are mostly active techniques, as well as stereo vision which measures depth from a single viewing position by comparing two images taken from slightly displaced positions.

Depth maps have been applied on canopies, where inferring a complete or detailed 3D structure is not necessary, such as employed by Ivanov et al. [107] and Müller-Linow et al. [108] who estimated the structural parameters of canopies based on top-view stereo imaging set-ups in maize and sugar beet, respectively, and as utilized by Baharav et al. [39] who measured the plant heights and stem widths in a sorghum canopy based on side-view depth maps.

Depth maps have also been applied on individual plants of which the leaves are planar and have an orientation more or less perpendicular to the viewing direction. Xia et al. [109] introduced the use of depth maps merely to provide a more robust segmentation of individual leaves of bell pepper plants where 2D RGB imaging would have had difficulty separating overlapping leaves. Chéné et al. [110] explored the use of depth imaging systems for leaf segmentation, as well as for the estimation of some 3D traits, such as leaf curvatures and leaf angles. Dornbusch et al. [10] used depth maps to monitor and analyze the diurnal patterns of leaf hyponasty, the upward movement of leaves in response to environmental changes, in thale cress. Depth map techniques can also be combined with other techniques: Li et al. [111] combined depth image data with 3D point cloud information to carry out in situ leaf segmentation for different kinds of plant species such as *Hedera nepalensis*, *Epipremnum aureum*, *Monstera deliciosa* and *Calathea makoyana*.

**Fig. 4:**
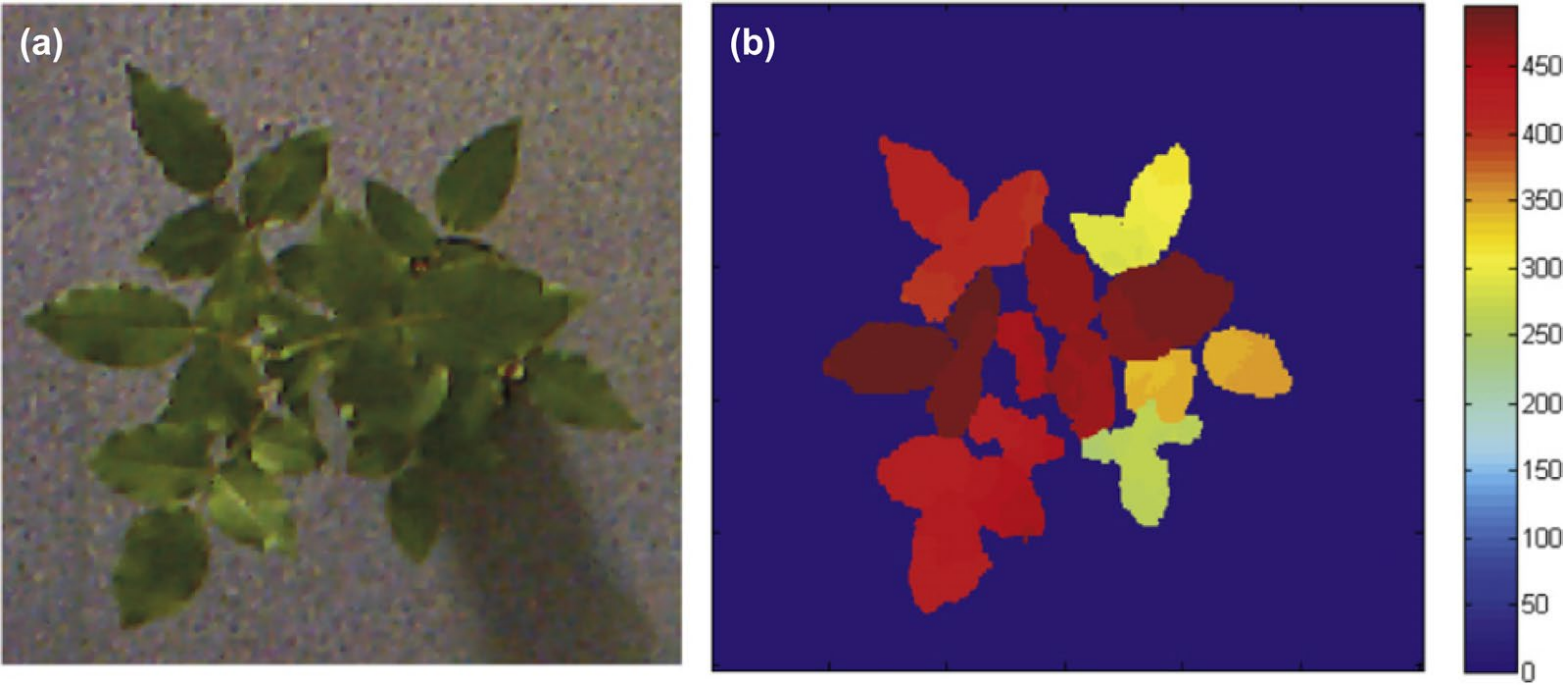
Top view RGB image of a rosebush (**A**), and depth map of the same rosebush scaled in mm with ground as reference, obtained by a Microsoft Kinect depth sensor (**B**), by [110]. The depth map allows to differentiate the different composite leaves, which would be much harder without depth information. Reprinted from [110], ©2015, with permission from Elsevier

Depth maps are particularly suitable for segmentation as illustrated in Fig. 4, but note that the segmentation and subsequent analysis of the segmented images will often suffer from occlusions, lacking the advantages of full 3D imaging. By covering the 3D scene from multiple angles and with overlapping images, 2.5D can be augmented to a real 3D point cloud with xyz-coordinates. Here, the Iterative Closest Point (ICP) algorithm [112] and variants thereof allow to match point clouds sampled from the overlapping depth maps.

#### Point cloud

A 3D point cloud is a set of points representing an object or surface. One of the advantages of the point cloud representation is that it includes depth information, thus working around the issue of occlusion among plant leaves [111]. Point clouds can be obtained in two ways: from active 3D image acquisition techniques, like image-derived methods, LiDAR, RGB-D cameras or synthetic aperture radar systems, or through (passive) 3D reconstruction from a set of different views from the scene [86, 113, 114]. Among the active methods, LiDAR point clouds are commonly used for point cloud segmentation applications and for trees (forests) [115].

Active image acquisition methods typically give rise to point clouds of relatively uniformly sampled points on the surface of the represented objects. The density of point clouds acquired through passive photogrammetric techniques, however, will often depend on the presence of detectable features on the surface of objects, because such techniques usually rely on finding corresponding sets of said points on multiple overlapping 2D images. This can result in point clouds where featureless parts of objects are less well represented, or in false points due to mismatches between features, especially when the scene contains repeated structures.

Point clouds do not directly provide information about the surface topology [20, 116], implying that it will be more challenging to accurately estimate an underlying surface or curve representation and to estimate traits related to the surface area, especially in the presence of noise, outliers or other imperfections. This will be even more difficult when dealing with the complex architecture of plants. Thus, the quality of the point cloud in conjunction with the nature of the plant architecture, will largely determine the available processing and analysis techniques.

Almost all of the techniques (both active and passive) result in a point cloud [18]. Cao et al. [14] generated a 3D point cloud by developing a low-cost 3D imaging system to quantify the variation in the growth and biomass of soybean due to flood at its early growth stages. Martinez et al. [13] created two dense point clouds using a low-cost SfM and an acquisition and reconstruction using an RGB-Depth Kinect sensor to examine the suitability of two low-cost systems for plant reconstruction, which was later used for the solid model creation. The model using SfM showed better results for the reconstruction of end-details and accuracy of the height estimation. However, use of RGB-D information was faster during the creation of the 3D models.

Ma et al. [43] produced a 3D point cloud by developing a 3D imaging approach to quantitatively analyze soybean canopy under natural light conditions. Most current systems provide information on the whole-plant level and there are only a few cases where information on the level of specific plant parts, such as leaves, nodes and stems, is given [2]. One such example can be found in Thapa et al. [117], who generated a 3D point cloud acquired with a LiDAR scanner to measure plant morphological traits, including the individual and total leaf area, the leaf inclination angle, and the leaf regular distribution of maize and sorghum.

#### Voxels

A 3D object may also be represented by a 3D array of cells, in which each cell (voxel) contains two possible values, indicating whether a voxel is occupied by the object or not. The most commonly used methods which result in such a representation are shape estimation methods [96] like Shape-from-silhouette (SFS) [118], space carving [98, 99], voxel coloring [97], and generalised voxel coloring [119, 120]. These passive methods rely on determining the visual hull, which is the largest possible shape that is consistent with the intersection of 2D silhouettes of an object projected into a 3D space.

If the plant structure is relatively simple, then these standard volumetric methods are relatively easy to implement, are fast, and produce good approximations. For example, Golbach et al. [101] used SFS to reconstruct tomato seedlings, and Kumar et al. [121] did the same for young maize and barley plants. Phattaralerphong et al. [122] also applied SFS to obtain voxel representations of tree canopies. Their goal was to measure traits such as tree height, tree crown diameter and canopy volume which don’t require very accurate 3D representations. Likewise, Kumar et al. [123] estimated maize root volume based on a voxel representation obtained by SFS.

However, if the scene is relatively complex, such as when multiple plant parts are overlapping, or the plant parts are very intricate, one may have to rely on less standard volumetric methods. For example, Klodt et al. [103] developed an optimization method which finds a segmentation of the volume enclosed by the visual hull by minimizing the surface area of the object subject to the constraint that the volume of the segmented object should be at least 90% of the volume enclosed by the visual hull. They applied their method for the volumetric 3D reconstruction of barley plants, and achieved an accurate 3D reconstruction of fine-scaled structures of the plant.

## 3D image processing

**Fig. 5:**
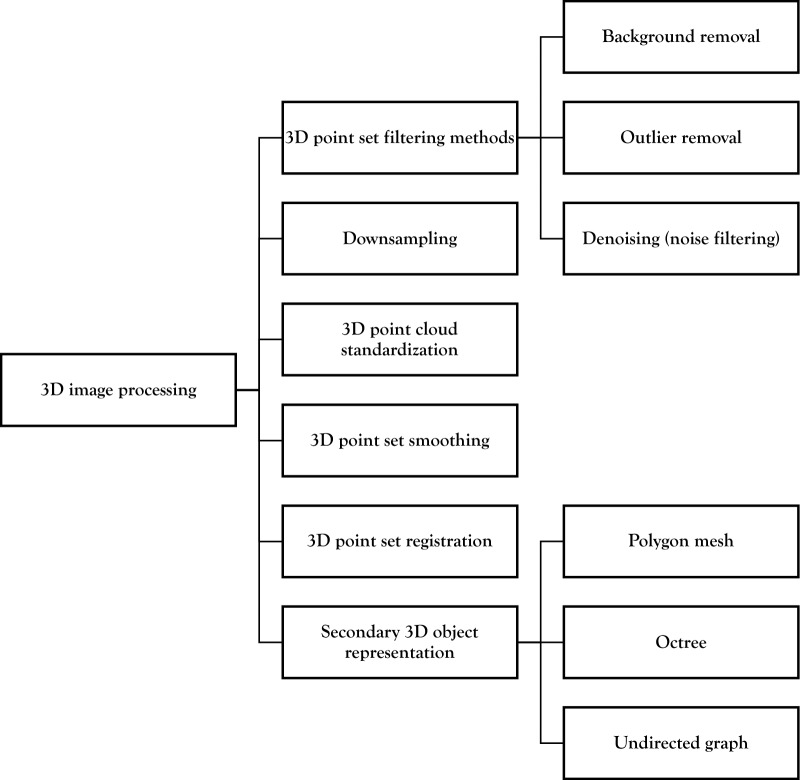
Overview of 3D image processing techniques

This section describes common techniques for the visualization, processing, and analysis of phenotyping data (in 3D point set form, as a 3D image, or in any other form), through transformations, filtering, image segmentation, and morphological operations. A full list of papers and plants using these techniques can be found in Table 3, under the header “3D Image Processing”. Moreover, an overview of the topics covered in this section is presented in Fig. 5.

### 3D point set filtering

Point sets contain noise stemming from different sources regardless of whether the point cloud was generated actively or passively (but passively generated point clouds are typically more noisy [86, 124]). Removing noise is an essential first step in the processing pipeline.

Actively generated point clouds typically suffer from limited sensor accuracy and measurement error due to environmental issues (illumination, material references and imperfect optics). For point clouds that are generated through computational reconstruction, imprecise depth triangulation and inaccurate camera parameters can give rise to significant geometry errors which can be classified in two types: outlier errors or positioning errors [86, 113, 114].

Moreover, the point cloud will often contain parts of the surrounding scene as well as wrongly assigned points, which need to be selectively removed, and “double wall” artefacts may result from small errors in the alignment of multiple scans, or from small movements during image acquisition. Finally, the initial size of the point cloud is often too large for further processing within a manageable time frame, requiring downsampling.

In plant phenotyping it is common to divide point set filtering into three different steps: background removal, outlier removal, and denoising [33, 125–127].

#### Background removal

When a point cloud is obtained through an active 3D acquisition method and doesn’t contain color information, usually efforts are made to capture as little of the surrounding scene as possible. If the point cloud still contains part of the surrounding scene, background removal can rely on the detection of geometric shapes such as planes, cylinders, or cones which may correspond to a surface, the main stem, or a pot, respectively. Points can then be discarded depending on the relative position to these features. Detection of geometric shapes is often done using the RANSAC algorithm [128]. For example, Garrido et al. [129] imaged maize plants in a field using LiDARs mounted on an autonomous vehicle, and used RANSAC to segment their point clouds into ground and plants. Liu et al. [130] used a variant of the RANSAC algorithm named MSAC to separate the soil from the original point cloud of maize.

When active 3D acquisition is combined with an RGB-camera or when a passive 3D acquisition method is applied, color information can be used for the removal of background points. The efforts employed in controlling lighting conditions during the 3D acquisition will determine whether one can rely on simple color thresholding or more complex clustering or classification methods to discriminate between plant and background, based on color. For example, Jay et al. [79] used clustering based on both height above ground and color to discriminate between plant and background points in point clouds of in-field crop rows of various vegetable species which were obtained by SfM. Ma et al. [43] extracted soybean canopies from background objects: point clouds were rasterized to depth images, after which the pixels of the soybean canopies were differentiated from those of the background by using spatial information in the depth images. Although color information can be useful for removing background points, plants often present ranges of similar colors and shapes, making it difficult to perform segmentation. To remedy this, Sampio et al. [15] developed a new technique using only (logarithmically transformed) depth information, and they show that accurate reconstruction results can be obtained for maize plants.

In the case of true background noise, this can be removed using a pass-through filter which limits the range of axes and removes the points outside the range. This approach can easily be combined with other filtering algorithms such as the minimum oriented bounding box (MOBB) algorithm [125].

#### Outlier removal

Two methods for outlier removal are regularly applied on point clouds: radius and statistical outlier removal. The radius outlier removal method counts the number of neighboring points within a certain specified radius and removes the points for which this number is lower than a specified minimum number of neighbors. In statistical outlier removal (SOR) the mean distance to the *k* nearest neighbors is calculated for each point. Points are removed if the mean distance surpasses a certain threshold which is based on the global mean distance to the *k* nearest neighbors and the standard deviation of the mean distances.

Li et al. [111] developed a novel 3D joint filtering operator by integrating a radius-based outlier filter that can separate leaves by removing sparse points for different kinds of plant species such as *Hedera nepalensis*, *Epipremnum aureum*, *Monstera deliciosa* and *Calathea makoyana*. Liu et al. [130] applied a MATLAB function (pcdenoise) to remove outliers from the point cloud of maize which are at least 0.3 SD away from the mean distance and then applied another MATLAB function (pcsegdist) to remove the larger outliers according to a Euclidean distance threshold of 5 mm. Sampaio et al. [15] and Chaivivatrakul et al. [38] used the same method to remove the outliers from the point clouds of maize plants.

#### Denoising (noise filtering)

Before applying further analysis steps it may be necessary to correct certain irregularities in the data, such as noise and “double walls” artefacts.

*Moving Least Squares (MLS)* This technique iteratively projects points on weighted least squares fits of their neighborhoods, thus causing the newly sampled points to lie closer to an underlying surface [131].

*Density-based spatial clustering of applications with noise (DBSCAN)* This algorithm was proposed by Ester et al. [132] and is a density-based clustering algorithm designed to discover clusters of arbitrary shape. Zermas et al. [82] used an algorithm based on DBSCAN to remove clusters that are smaller than a certain threshold and located further away than a fixed distance from other clusters, and applied this algorithm to maize plants.

*Spatial Region Filter* This filter works by means of region specifications which consists of one or more region expressions (geometric shapes) combined according to the rules of Boolean algebra. It is used for plants such as *Epipremnum aureum*, *Monstera deliciosa*, *Alathea makoyana*, *Hedera nepalensis* and maize in the works of Wu et al. [133] and Li et al. [111].

*Color filtering* Lou et al. [9] used a color filter to remove noisy points from a 3D point cloud. They acquired images from the plant against a dark background, and found that background noisy points were mostly colored dark, whereas points belonging to the plant were shades of green.

### Downsampling

Reducing the number of points needs to happen in a way which minimizes loss of information about surface and topology of the sampled object. The most regularly used method for point cloud downsampling is the voxel-grid filter. Here the point cloud is divided into a 3D voxel grid and points within each voxel are replaced by the centroid of all points within that voxel [9, 111, 130, 134, 135].

An alternative method, which makes use of random sampling and which is also designed to retain key structures in the point cloud, is the dart throwing filter [136], where points from the original point cloud are sequentially added to the downsampled point cloud if they don’t have a neighbor in the output point cloud within a specified radius.

### 3D point cloud standardization

Point cloud standardization [15] refers to the process of adjusting the resolution of the point cloud according to the object in the scene, where, for example, objects with larger proportions can be described using a lower density of points while smaller objects are described using higher point densities. The result is a point cloud from which extraneous detail has been removed, resulting in a lower amount of data while keeping essential object features.

Sampio et al. [15] presented a point cloud standardization procedure in which an octree data structure was used to hierarchically group cloud points into voxels according to a predefined resolution, with each voxel described by a single point in the group (e.g., the centroid).

### 3D point set smoothing

The raw imaging data acquired from optical devices such as laser scanners always contains noise [137], which must be taken into account during subsequent post-processing.

One pervasive source of error for ToF cameras is the so-called wiggling error [138–141], which alters the measured distance by shifting the distance information significantly towards or away from the camera depending on the surface’s true distance [138]. The wiggling error can be addressed by using bilateral smoothing, a non-linear filtering technique introduced by Tomasi and Manduchi [142] for edge-preserving smoothing [137].

Sampaio et al. [15] used the bilateral smoothing technique for smoothing the cloud points of maize plants in two steps: smoothing normals and points repositioning based on the adjusted normals, while the estimation of the normal vector for each point is performed using the Principal Component Analysis (PCA) technique. Ma et al. [125] used a bilateral filter to smooth the point cloud of rapeseed while preserving the edge features of the point cloud. He and Chen [141] implemented an error correction for ToF sensors based on a spatial error model and showed that this approach performs better in comparison to the calibration method in [143] or the distance overestimation error correction methods in [144].

### 3D point set registration

Many imaging methods give rise to more than one 3D point cloud, for instance when observing a plant from different viewing angles, and these point clouds need to be reconciled with one another into a single coordinate system, a process known as 3D point cloud registration [125, 145]. In the case of two 3D point clouds this process is known as pairwise registration, and is studied extensively in the computer vision literature [145–149]. For pairwise registration, one set of points is typically kept fixed and denoted as the “target”, while the other is designated as the “source”. The goal is then to iteratively move the points of the source towards the target, while keeping the total amount of motion or deformation limited.

Broadly speaking, there are two categories of registration algorithms: rigid and non-rigid. Rigid point registration methods estimate a rigid body transformation (translation and rotation) of the source onto the target, and are usually easier to handle since they involve fewer parameters [150]. Chief among the rigid registration algorithms is the Iterative Closest Point (ICP) algorithm [112, 151], which alternates between associating nearby points in the source and the target, and estimating an optimal rigid body transform [152]. Many variants and improvements of the ICP algorithm exist [42, 153, 154], incorporating additional sources of geometric information (e.g., depth), or optimizing for point cloud data from specific acquisition devices such as the Kinect. Rigid point registration methods have been applied extensively for plant phenotyping. Wang and Chen [155], for example, developed an improved ICP algorithm that is more suitable for registering 3D point clouds from different directions using a turntable. They used a rotation matrix and a translation vector to process the relationship between adjacent point clouds and then applied the ICP algorithm. They applied their method on pepper plants and showed that the improved ICP has a better result in comparison to traditional ICP.

Rigid point registration algorithms perform well for rigid structures that are already somewhat aligned, but tend to yield poor results for the registration of deformable structures, such as non-rigid, thin plant structures [156]. Non-rigid registration techniques allow each point of the point cloud to move independently while penalizing large deformations. Moreover, the presence of noise and outliers may complicate the search for an optimal registration, rigid or otherwise. To accommodate noise, Jian and Vemuri [157] represent the input point sets as Gaussian Mixture Models (GMM) and reformulate the problem of image registration as one in which the distance between two GMMs is minimized, achieving good performance in terms of both robustness and accuracy [158]. It is worth noting that this approach can be applied to both rigid and non-rigid registration methods. The GMM approach is developed further in the Coherent Point Drift (CPD) algorithm of Myronenko et al. [159], where additionally the centroids of the Gaussians of one point set are constrained to approximately move together, so that the topological structure of the point cloud is preserved.

In the context of plant phenotyping, Chaudhury et al. [156] developed a two step method that achieved a better fit than CPD in case of registering multiple scans. This method starts with aligning the scans and then registers a single scan to the average shape, constructed from all other scans, and updates the set to include the newly registered result. They applied their method on thale cress and barley plants. Ma et al. [125] used the Fast Point Feature Histogram (FPFH), explained in the "Clustering-based methods" section, for rough registration to register multiple neighboring point clouds into a single point cloud and an ICP algorithm for fine alignment. Teng et al. [33] developed an improved ICP and applied it on rapeseed plants and then compared it with classic ICP. Apart from being computationally more effective, the new method also succeeds in registering point clouds with large differences in angles, for which registration fails using the classical ICP.

**Fig. 6:**
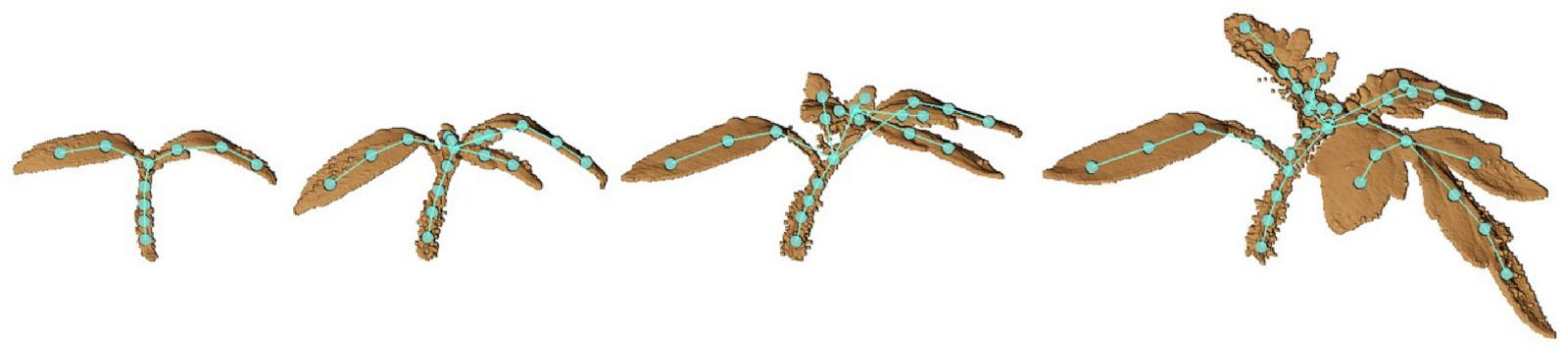
Time series of a tomato plant scanned in various days together with the extracted skeleton. Reprinted from [160] under the terms of the Creative Commons Attribution 4.0 International License (http://creativecommons.org/licenses/by/4.0)

Lastly, one of the most challenging tasks is registering 3D point clouds of the plants over time and space [34]. Performing analysis on the time-series plant point cloud data, one needs to come up with techniques that associate the point cloud data over time and register them against each other. The plants changing topology, as well as non-rigid motion in between plant scans make plant registration over an extended period of time very challenging [160]. Chebrolu et al. [34] and Magistri et al. [161] tackled the complexity of registering plant data over time by exploiting the skeleton structure (see "Skeletonization" section) of the plant to obtain correspondences between the same plant parts for the scans on different days (Fig. 6). To aid with the development of new algorithms for point cloud registration among other things, Schunk et al. [160] compiled Pheno4D, a large scale spatio-temporal dataset of point clouds of maize and tomato plants.

### Secondary 3D object representations

Depending on subsequent analysis methods it may be advantageous to convert the 3D representation into one of the below secondary representations.

#### Polygon mesh

A polygon mesh is a 3D representation composed of vertices, edges and faces which define the shape of an object. The construction of a polygon mesh as an intermediate step in the analysis of a 3D representation of a plant may, for example, facilitate the calculation of leaf surface areas, or the segmentation into individual organs.

Polygon meshes are commonly constructed from voxels using the Marching Cubes algorithm [162], or from point clouds using $\alpha$-shape triangulation [163]. However, mesh generation requires precise point clouds or voxel representations, and the intricate and non-solid nature of the plant architecture makes that generating polygon meshes on a whole plant is often not feasible. More often, surface fitting is performed on individual leaves, after segmentation, or different surface fitting methods are applied to different plant organs.

Paproki et al. [164] constructed meshes of cotton plants from point clouds obtained by multi-view stereo, and performed their phenotypic analysis based on this representation. They could obtain measurements of individual leaves and track them through time. McCormick et al. [165] also based their measurements of shoot height, leaf widths, lengths, areas and angles in sorghum on the generation of a mesh from point clouds obtained through laser scanning. Chaudhury et al. [156] generated a mesh on complete thale cress point clouds by $\alpha$-shape triangulation to determine total surface area and volume.

#### Octree

An octree [166] is a tree-like data structure, in which a 3D space is recursively subdivided into eight octants if the parent octant contains at least one point. In this way, increasing tree depths represent the point cloud in increasing resolutions. Such a representation can avoid memory limitations when points need to be searched within a large point cloud.

There are various algorithms for clustering and skeletonization which exploit the octree data structure, and which are suitable for plant phenotyping, such as CAMPINO [167] and SkelTre [168].

Duan et al. [169] used octrees to divide point clouds of wheat seedlings into primary groups of points, after which these primary groups were merged manually to make them correspond to individual plant organs. Scharr et al. [104] developed an efficient algorithm for voxel carving on banana seedlings and maize, which directly outputs an octree representation. Zhu et al. [170] used an adapted octree to reconstruct the surface of the 3D point cloud of soybean plants.

#### Undirected graph

An undirected graph is a structure composed of vertices connected by edges. Edges are assigned weights corresponding to the distance between the connected points. Useful algorithms such as Dijkstra’s algorithm to calculate shortest paths [171], Minimum Spanning Tree [172], and graph-based clustering methods such as spectral clustering [173] use undirected graphs as input.

An undirected graph can be constructed from a point cloud by connecting neighboring points to the query point. Neighbors can be selected based on a certain radius *r* around the query point, or the *k* closest neighbors can be selected. If *r* or *k* are chosen too high, many redundant edges will be formed, whereas if they are too low, crucial ones may be missed.

Hétroy-Wheeler et al. [174] converted the point clouds of various tree seedlings, obtained through laser scanning, into an undirected graph and used this as the basis for spectral clustering into plant organs. To avoid redundant edges and thus speed up the computation of subsequent steps, while at the same time not miss any relevant edges, they pruned the edges which have neighbors within a certain radius *r*, based on the angles between edges.

## 3D image analysis

**Fig. 7:**
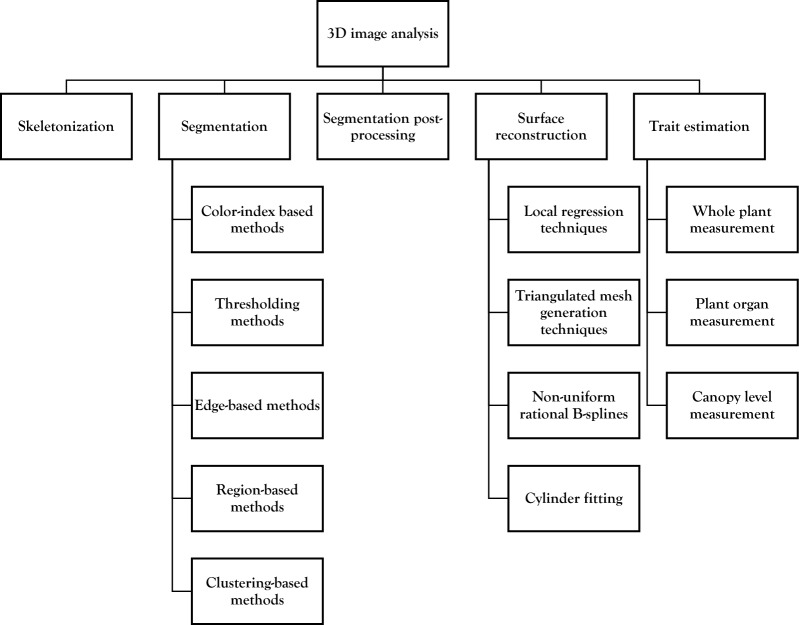
Overview of 3D image analysis techniques

The above processing steps are merely a transformation of the original 3D representation as preparation for subsequent analysis steps. During these analysis steps, specific additional information is extracted from the 3D representation. A full list of papers and plants using these techniques can be found in Table 3, under the header “3D Image Analysis”. An overview of the topics covered in this section is presented in Fig. 7.

### Skeletonization

Skeletonization is the process of calculating a thin version of a shape to simplify and emphasize the geometrical and topological properties of that shape, such as length, direction or branching, which are useful for the estimation of phenotypic traits. A plethora of algorithms has been developed to generate curve skeletons. These techniques make use of different theoretical frameworks such as topological thinning or medial axes. For a review of methods in the context of plant images, see Bucksch and Alexander [175], and for a more general overview of methods, see Cornea et al. [176]. Skeletonization usually results in a set of voxels or points that in a final step are connected into an undirected graph, and on which subsequent analyzes can be performed.

A number of studies have proposed algorithms to model the 3D structure of trees by skeletonization, either for the purpose of phenotyping or for computer graphics. In Livny et al. [177] and Mei et al. [178] skeletonization of point clouds of trees obtained by terrestrial LiDAR scanning was performed, not to build an accurate 3D representation of the trees for phenotyping, but to generate models of trees with a credible visual appearance for computer graphics. Despite this different perspective, both provide skeletonization methods which should also be suitable for plant phenotyping, when excluding the processing steps which only serve to enhance the visual appearance of the 3D models.

Bucksch et al. [168] developed a fast skeletonization algorithm, and obtained good results comparing the distributions of skeleton branch lengths with and manually measured branch lengths [179]. While the method is fast, it performs less well for point clouds with varying point densities, and is likely to face difficulties with plants other than the leafless trees which they studied.

Coté et al. [180] constructed 3D models of pine trees by skeletonization to obtain realistic models in order to study reflected and transmitted light signatures of trees, by ingestion into a 3D radiative transfer model. Here again the goal was not to obtain direct phenotypic measurements of individual trees, but to study indirect radiative properties which depend on the tree canopy structure. To this end, they generated plausible tree canopy structures from a skeleton structural frame defining the trunk and first-order branches only. The skeletonization method employed to create this structural frame uses a method proposed by Verroust and Lazarus [181] based on the use of Dijkstra’s algorithm applied on an undirected graph.

The aforementioned method assumed that cloud points are sampled uniformly or nearly uniformly. To handle point clouds with inconsistent density and outliers, Delagrange et al. [182] developed PypeTree, a software tool for the extraction of skeletons of trees that allows the user to manually adjust a reconstructed plant skeleton.

Ziamtsov and Navlakha [183] improved upon PypeTree [182] and the methods of Verroust and Lazarus [181] and Bucksch and Alexander [175] by using information about the curvature of the plant skeleton. They did so by adding two new features to detect plant tips more accurately and independently of connected components or level size, and to enhance root selection. They apply their method to extract a skeleton graph of tomato and benth plants.

Lou et al. [9] adopted a method developed by Cao et al. [184] based on Laplacian contraction and applied this method on thale cress (rosette and in flowering stage), Physalis sp., maize, Brassica sp., and wheat. They first segmented the leaves and after removing them from the point cloud, they applied their method to the modified version of the point cloud. This method proved to be robust to noise and produced a well connected skeleton.

The extracted 3D reconstructions usually contain in the order of millions of points which imposes significant computational demands on subsequent processing steps. Therefore, another application for skeletonization is to provide a more parsimonious representation of a plant structure so that further processing can be done more efficiently. For example, Zermas et al. [82] developed a skeletonization algorithm starting from 3D point cloud data, which is split into thin slices of equal height. A per-slice clustering is then performed to find cluster centroids that best represent the neigboring points, and these cluster centroids are retained in the thinned-out skeleton. They applied this method on maize plants.

Chaudhury and Godin [185] proposed an algorithm based on stochastic optimization to improve coarse initial skeletons that were obtained with different skeletonization algorithms. They applied the proposed algorithm on real world and synthetic datasets contains different varieties of plants including cherry, apple tree, and thale cress plants. In contrast to other techniques, their method is more faithful to the biological origin of the original point cloud data.

Wu et al. [133, 186], on the other hand, used an iterative shrinkage process to contract the point cloud of a maize plant by using the classical restricted Laplace operator.

The 3D analysis of the branching structure of root systems is another application which has been approached by skeletonization. For example, Clark et al. [187] present a software tool for the 3D imaging and analysis of roots. Here, a thinning algorithm is applied on voxel representation obtained by SFS.

Despite its usefulness for the estimation of certain traits, skeletonization has rarely been applied to the phenotyping of herbaceous plant shoots. This may be because of difficulties when applying skeletonization on objects with more diverse topographies, such as in the presence of broad leaves, and when there are more occlusions. Chaivivatrakul et al. [38] performed a medial axis-based skeletonization of the relatively simple structure of young maize plants to obtain leaf angles, but they found that that particular skeletonization method didn’t perform well compared to plane fitting through leaves.

### Segmentation

Image segmentation is the process of dividing an image into parts based on the problem needs [16]. In plant phenotyping, segmentation of the 3D representation into individual plant organs is a difficult and critical step in the process of obtaining plant organ measurements. There is no standard approach that will work in the majority of situations. The application of any one approach will largely depend on the plant morphology, as well as the quality of the 3D representations.

There are several existing techniques which are used for image segmentation and all these techniques can be approached from two basic approaches of segmentation: region-based and edge-based approaches [188, 189]. The most popular techniques and their application in plant phenotyping are listed below [16, 188, 190]. A comparison of the different segmentation techniques is presented in Table 4.

**Table 4:** A comparison of segmentation techniques

Segmentation method	Principle	Advantages	Disadvantages
Clustering-based	Segments the image into clusters consisting of pixels with similar characteristics	(1) Elimination of noisy spots (2) Typically obtains homogeneous regions	(1) Sensitive to noise (2) Hard to find initial parameters
Color-index-based	Makes a distinction between foreground and background values based on a scalar value (e.g., green channel)	(1) Simple to implement (2) Low computational cost (3) High efficiency	(1) Omitting spatial information by only considering pixel intensities (2) Sensitive to noise
Edge-based	Detects edge points based on sudden changes in intensity and generates edge segments by grouping edge points together	(1) High accuracy in edge positioning (2) High speed	(1) No guarantees about continuity and closure of edges (2) Less suitable for images with many edges
Region-based	Divides the point cloud into different clusters based on local smoothness and curvature characteristics or on the presence of features at a certain scale	(1) Effective for complex images (2) High accuracy in images with high contrast between regions (3) Generally good performance in noisy images	(1) Complicated algorithm (2) Computationally intensive
Threshold-based	Divides pixels into groups based on their intensity relative to a given value or threshold	(1) Simple to implement (2) Low computational cost (3) High efficiency	(1) Depending only on the pixel gray value without considering spatial details (2) Sensitive to noise

#### Color-index based methods

A common method for segmenting the plant from the background is color index-based segmentation [8]. In this approach, a 3D color value is converted into a scalar (grayscale) value, so that there is a pronounced distinction between foreground and background values.

Ge et al. [191] used color index-based segmentation on maize plants in which the image was transformed to a single color-band image using a nonlinear transformation emphasizing the green channel and suppressing the effects of different illuminations. Choudhury et al. [192] used color-based segmentation in hue, saturation, and value (HSV) color space for a holistic and components-based phenotyping of maize plants.

#### Thresholding methods

Assuming strict conditions as to the composition of the scene, the majority of algorithms in plant phenotyping usually employ thresholding-based approaches in one or multiple channels [193–195]. Gray-level thresholding is the simplest segmentation process and using a threshold can segment objects and background [16].

Minervini et al. [196] used a binary segmentation of thale cress and tobacco plants as the first step. Xia et al. [109] applied an RGB thresholding method to field images of paprika plants to eliminate the background.

#### Edge-based methods

A large group of methods performs segmentation based on the information about edges in the image. Edge detection algorithms usually work in two steps: first, points belonging to an edge are detected based on quick changes of the intensity around the point. Then, edge segments are generated by grouping points inside the boundaries extracted by edge detection [16, 188, 197, 198]. This method is simple and fast, but is more suitable for 2D images rather than 3D point clouds and often delivers disconnected edges which cannot be used to identify closed segments [115, 189, 198].

Lomte and Janwale [199] provided a brief review on plant leaves segmentation techniques including edge-based techniques on 2D images. Some works on edge-based segmentation on 2D images can be found in [200] on thale cress, and [201] on orange fruits, [202] on pigweed, purslane, soybean, and stinkweed.

#### Region-based methods

Segmentation results from edge-based methods and region-growing methods are not usually the same. However, region-growing techniques are generally better in noisy images, where it is difficult to detect borders between regions of the image with similar characteristics, such as intensity or color [16].

Liu et al. [130] developed a three-phase segmentation procedure to segment maize plant organs based on a skeleton and a region-growing algorithm. First, they processed the denoised point clouds of each plant using a Laplacian-based method [184] and generated plant skeleton points. They then applied a region-growing algorithm proposed by Rabbani et al. [197] to classify point cloud clusters.

Miao et al. [203] applied a median-based region-growing algorithm [204] to segment the stem points of the maize plant. Their algorithm is a region-growth method tailored specifically to maize and is able to segment stem and leaf instances in sequence, working upwards from the bottom of the plant.

Region-growing algorithms divide the point cloud into different clusters based on local smoothness and curvature characteristics or on the presence of features at a certain scale. Typically, these characteristics vary across a wide range of values for plant point clouds, and a threshold that works for one plant type or organ may not be appropriate for another. To address this, Huang et al. [205] developed a multi-level region-growing segmentation to find a suitable adaptive segmentation scale for different input data. They applied the proposed method to perform individual leaf segmentation of two leaf shape models with different levels of occlusion. They compared their proposed method with two widely used segmentation methods (Euclidean clustering and facet region-growing methods) and showed that the proposed method has the highest measurement accuracy.

**Fig. 8:**
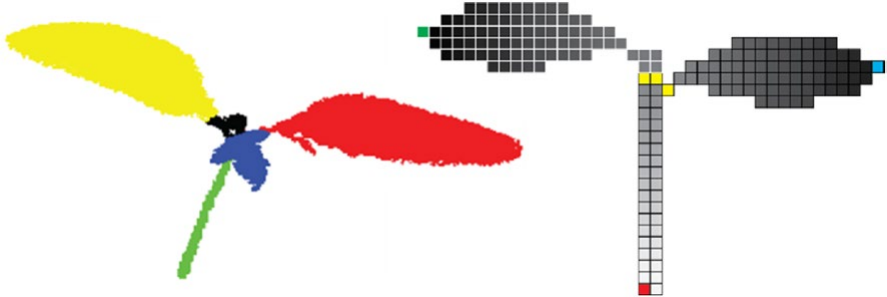
Segmentation of a voxel grid representation of a tomato seedling in stem (green) and individual leaves (colored) (left), and schematic illustration of the stem-leaf segmentation algorithm (right), as used by [101]. The structure is filled from the bottom (red point). As long as neighboring points are close together in space, they are treated as stem. Once they spread out, the end of the stem (yellow points) is marked. The last point additions correspond to leaftips (green and blue points). Reprinted under the terms of the Creative Commons Attribution 4.0 International License (http://creativecommons.org/licenses/by/4.0)

Golbach et al. [101] performed a segmentation of stem and leaves on a voxel representation of tomato seedlings. They used a breadth-first flood-fill like algorithm whereby the structure is iteratively traversed along neighboring voxels starting from the lowest point in the voxel representation. As the algorithm traverses the main stem all added points are located closely together, but at the point of the first side branches newly added voxels are located further apart. If this distance exceeds a certain threshold, the iteration can be treated as the end of the stem. Leaf tips were detected as the last voxel additions after the flood-fill algorithm progressed past the end-point of the main stem. This approach is illustrated in Fig. 8.

Klodt and Cremers [103] segmented their volumetric 3D models of barley into two regions based on the eigenvalues of the second moment tensors of the surface. These provide information on the gradient directions of the shape, and allow to discriminate between long, flat, or structures with no dominant direction. This approach resulted in a discrimination between the distal parts of leaves and the rest of the plant. The obtained segmentation then allowed for automated leaf quantification, by counting the number of connected components corresponding to the distal parts of the leaves.

The last two examples of segmentation algorithms [101, 103] are highly customized towards particular plant morphologies. The former makes use of the opposite position of the cotyledons of young dicot seedlings, while the latter depends on plants with a rosette-like arrangement of narrow leaves. The advantage of such highly customized algorithms is that they can be better tailored towards efficiency for use in high-throughput applications.

Choudhury et al. [99] used a technique called voxel overlapping consistency check with point cloud clustering techniques to divide the 3D plant voxel-grid of maize and cotton plants into three components based on the structure of the plants: stem, leaves and top leaf cluster to compute component phenotypes.

On polygon meshes, there are two common approaches for segmentation: the fitting of shape primitives such as planes, spheres and cylinders [206]; and region-growing from seed points on the mesh surface, constrained by changes in curvature which correspond to sharp edges [207, 208].

Paproki et al. [164] applied a hybrid segmentation pipeline based on both approaches. First they obtained a coarse segmentation of meshes of cotton plants into different leaves and the main stem using constrained region-growing. After that, more refined segmentation of the main stem region into internodes, and petioles branching off from the main stem, was performed using cylinder fitting.

Nguyen et al. [209] were mainly interested in segmentation into individual leaves and the stem, and applied region-growing constrained by curvature from seed points which were determined to belong to large flat regions based on pre-computed curvature values. They did this on a plastic model of a dicotyl plant, and their method allowed them to measure length, width, perimeter, and surface area of all the leaves.

#### Clustering-based methods

Clustering-based techniques segment the image into clusters consisting of pixels with similar characteristics [210, 211]. The most used techniques in this category in the plant phenotyping domain are discussed below.

*Topological and morphological feature-based:* Miao et al. [212] presented an automatic stem-leaf segmentation method for maize plants, which was able to extract the skeleton of a point cloud directly, and uses topological and morphological features to identify the number and category of organs. They generated a coarse segmentation based on the plant skeleton and used this result to classify the points into stem-leaf clusters. They showed that their method achieved a high segmentation accuracy.

*Mean shift:* Mean shift clustering was originally introduced by Fukunaga and Hostetler [213] and revisited after 20 years by Cheng [214]. This algorithm has been widely applied in image segmentation and object tracking [215, 216] and consists of an iterative procedure that shifts each data point to the average of data points in its neighborhood by using kernel density estimation [109]. Xia et al. [109] applied the mean shift algorithm to segment plant leaves and background objects in a depth image. Since depth data represent the coordinates of objects in 3D space, plant leaves and background objects could be separated in terms of discontinuity in depth.

*Spectral clustering (graph-based):* Spectral clustering goes back to Donath and Hoffman [217] and is a set of clustering techniques that takes connectivity between points in an undirected graph into account. Its main advantage is that it is straightforward to implement and can be solved efficiently by standard linear algebra methods [218]. Points are projected into a lower-dimensional embedding which maintains distances between connected points as much as possible. Next, a standard clustering technique is usually applied on this lower-dimensional embedding. When applying the spectral dimension reduction on a graph of a branching structure, such as a plant, this same branching should be recognisable in the lower-dimensional embedding, while other morphological features will be suppressed. A exhaustive introduction to spectral clustering can be found in the tutorial of von Luxburg [218].

**Fig. 9:**
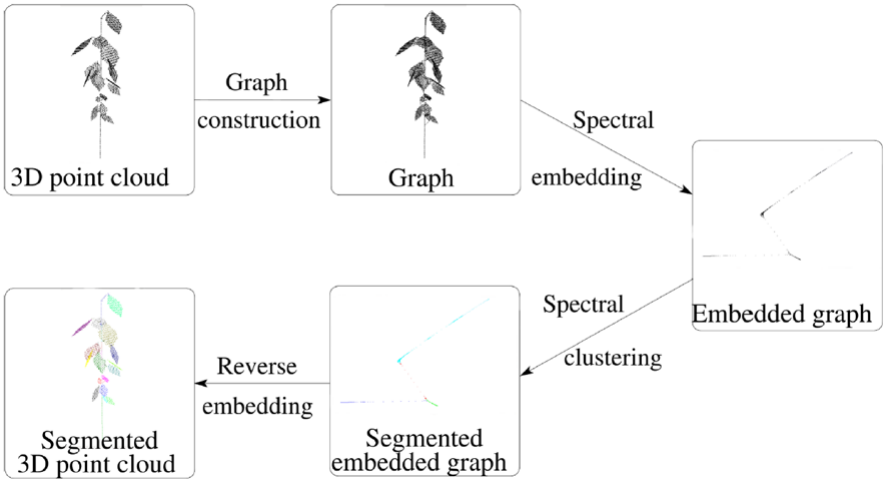
Illustration of the spectral clustering approach used by [174]. The point cloud obtained by laser scanning is converted into a graph representation, after which spectral embedding finds intrinsic plant directions, which are decomposed in the principal plant axes. These correspond to elementary units such as leaf blades, petioles, and stems. Reprinted by permission of the publisher Taylor & Francis Ltd, (http://www.tandfonline.com) and the authors

Hétroy-Wheeler et al. [174] and Boltcheva et al. [219] made use of this property to segment point clouds of poplar seedlings into individual leaves and their stems. They identified segments in the branching structure of the lower dimensional embedding, which correspond to the plant parts in the original point cloud of the tree seedling (Fig. 9).

Zermas et al. [82] applied an algorithm named Randomly Intercepted Nodes (RAIN) to segment the maize plant. Based on this algorithm, a rain drop that falls on any part of the plant has to glide on top of the plant’s surface before it reaches the ground and can only take two possible routes: fall over the edge of a leaf, or follow the stem closely until it reaches the plant base. By simulating and analysing the trajectories of hundreds of randomly placed rain drops, they were able to perform plant segmentation and extract other phenotypical characteristics. The selection of each next point was based on a few simple rules affected by gravity. Since most of the random drops encountered at a given moment an already visited point, at which time their route was prematurely ended, the number of points that were considered as potential path candidates was severely reduced. Like other algorithms, this algorithm has limitations as well. In dense canopies, for example, drops that visit a tall plant overshadowing a smaller plant may miss the smaller plant partially or completely.

Lou et al. [9, 220] proposed a spectral method for 3D mesh segmentation of CAD models. They showed that their method is applicable to diverse plants with varied structure, size and shape, and they applied their method on plants including thale cress, Brassica sp., oat, maize, Physalis sp. and wheat. However, this method cannot always generate meaningful and accurate segmentation results for plants with curved leaves, or with tiny side-branches at the top of the plant, or at junction points in the plant skeleton.

*Saliency features (Surface-based clustering):* The ordered eigenvalues resulting from eigendecomposition ($\lambda _0 \le \lambda _1 \le \lambda _2$) can be used directly as features for clustering or classification, because the relative size of the eigenvalues provides information about the shape of the local distribution of points: if points are scattered with no preferred direction, $\lambda _0 \simeq \lambda _1 \simeq \lambda _2$; if points are distributed along one axis, as would be the case for stems, $\lambda _2 \gg \lambda _0, \lambda _1$; and in the case of a planar surface, as for leaves, $\lambda _1, \lambda _2 \gg \lambda _0$. Therefore linear combinations of the eigenvalues, called the saliency features, could be used as features: *scatter-ness* ($\lambda _0$), *linear-ness* ($\lambda _2 - \lambda _1$), and *surface-ness* ($\lambda _1 - \lambda _0$).

These features can also be expressed as curvature and directionality, defined as $\lambda _0/(\lambda _0 + \lambda _1 + \lambda _2)$ and $\lambda _2/(\lambda _0 + \lambda _1 + \lambda _2)$, respectively. Points belonging to flat regions such as leaves will have a low curvature in their neighborhood, while linear features have a high directionality.

Dey et al. [221] used saliency features and color to segment point clouds of grapevines obtained through SfM [222] into branches, leaves and fruit. They calculated saliency features at 3 spatial scales and concatenated color in RGB to obtain a 12-dimensional feature vector for classification. Moriondo et al. [223] also used SfM to obtain point clouds of the canopy of young olive trees. They used saliency at one spatial scale and color features to segment the point clouds into stems and leaves using a Random Forest classifier. Li et al. [48] used curvature to discriminate between flat leaves and linear stems. They achieved a spatially coherent unsupervised binary classification via Markov Random Fields.

**Fig. 10:**
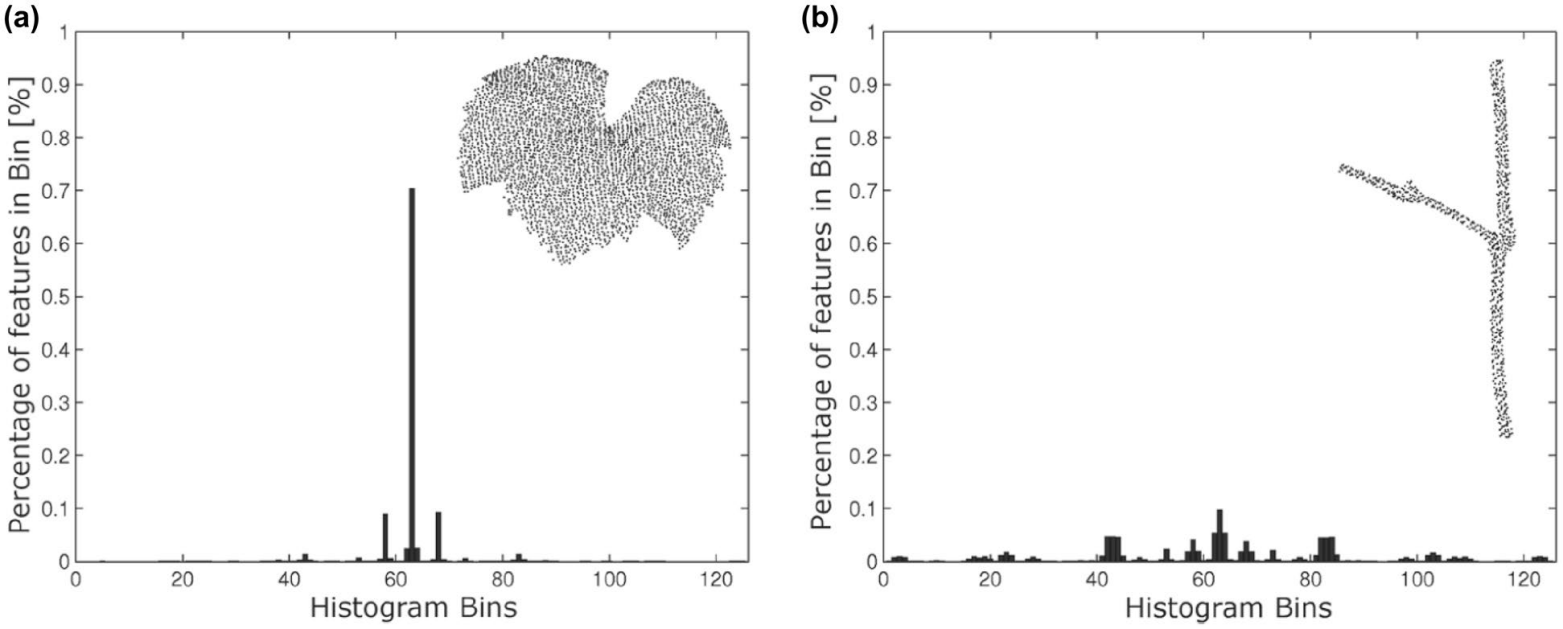
Point Feature Histograms for the laser scanned point cloud of a grapevine leaf (**a**) and of a grapevine stem point cloud (**b**), by [35]. Reprinted under the terms of the Creative Commons Attribution 4.0 International License (http://creativecommons.org/licenses/by/4.0)

*Point feature histograms:* Local features such as as surface normals or eigenvalues use only a few values in the neigborhood of a point. Point Feature Histograms (PFH) [224], and its more efficient variant Fast Point Feature Histograms (FPFH) [225], can be used for a more complete description of the neighborhood of a point. They are based on the angular relationships between pairs of points and their normals, within a radius *r* around each query point. These values, usually 4 angular features, are then binned into a histogram, and the histogram bins can be used as features in a clustering or classification algorithm. Figure 10 illustrates the difference in the PFHs between point clouds with different surface properties, such as of a laser scanned grapevine leaf and grapevine stem.

Because of their higher information richness, PFH depend on relatively precise and accurate representations of the plant organ surfaces and shapes, which usually will be obtained by active 3D acquisition techniques such as laser scanning. They have been used as features of high-precision point cloud representations of grapevine, wheat, and barley obtained by laser scanning [30, 35, 226]. Sodhi et al. [227], however, used less precise point clouds of sorghum plants obtained by multi-view stereo imaging, and could still obtain robust segmentations of leaves and stems because the shapes of plant organs in sorghum are relatively easily differentiated.

### Segmentation post-processing

A common post-processing step to improve the spatial consistency of class labels is to apply a fully connected pairwise Conditional Random Field (CRF) [228], which takes the spatial context into account and which can greatly improve segmentation results.

**Fig. 11:**
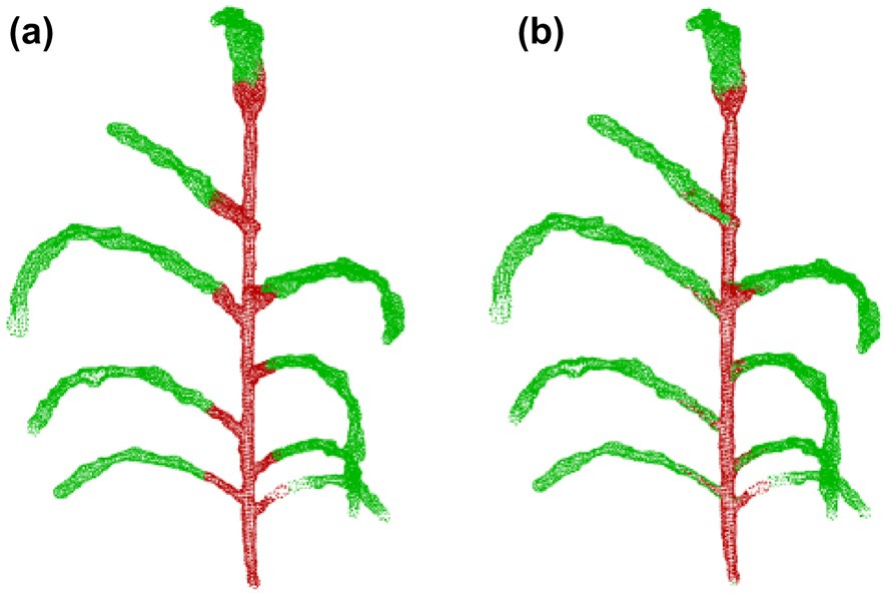
Segmentation obtained by SVM on FPFHs before **a** and after **b** post-processing with CRF by [227]. CRF corrects leaf false negatives near stem/leaf intersections, by minimizing label differences across neighbors with similar surface normals. ©2017 IEEE, reprinted with permission from the authors

Dey et al. [221] and Sodhi et al. [227] applied such a CRF as post-processing of segmentations based on saliency features and PFH for grapevine and sorghum plants, respectively. The effect of such post-processing is illustrated in Fig. 11.

### Surface reconstruction

In point clouds, surface reconstruction can be an aid for segmentation, or can serve as a preliminary step before the final measurement of individual plant organs.

Once the point cloud has been segmented, reconstruction of the points on the plant organ surface and the edges can be tackled in different ways, via surface fitting and edge fitting, respectively. Surface fitting can be done by fitting geometric primitives such as cylinders and planes, or flexible surfaces such as non-uniform rational B-splines (NURBS). Although surface fitting can generate a smooth surface, it can also result in serrated lines for the edges. Constructing the edges needs the edge points to be detected and then fitted separately by using, for example, 3D splines, which offer a degree of smoothness. As surface edges are typically noisy, detecting the constituent points of the edge directly can be difficult [229].

#### Local regression techniques

Least squares methods are a classic tool for surface fitting [230, 231]. However, applying least squares directly can generate a overly smooth surface that loses certain local details of the surface, like leaf structures. Hence, applying a method that uses local information may be more suitable for reconstructing the surface and capturing local details [229]. MLS (see also "Denoising (noise filtering)" section) is widely used for generating a surface for data points [232], and constructs and evaluates a local polynomial continuously over the entire domain instead of constructing a global approximation. This method can thus be viewed as a local regression method.

Zhu et al. [229] used another local regression method called Locally Estimated Scatterplot Smoothing (LOESS) which can reconstruct a continuous surface even with the presence of the discontinuity of leaf points and is similar to MLS. They used this method for maize plants and compared it with Poisson and B-spline methods, showing that this method can generate smoother leaf surfaces with smaller normal variances.

#### Triangulated mesh generation techniques

Triangulation for plant structures is challenging due to the presence of thin branches. Delaunay triangulation is typically used for modeling a surface but does not generate good results for plant structures [156].

Sampaio et al. used the Advancing Front algorithm [233, 234] based on Delaunay triangulation but with higher performance in terms of accuracy and quality. They applied this algorithm in the first phase of surface reconstruction for maize plants. Chaudhury et al. [156] used the $\alpha$-shape algorithm for triangulation on barley and thale cress plants and showed that it worked well when its parameters were properly tuned. Zhu et al. [229] applied the Delaunay triangulation algorithm [235] after surface fitting on maize and rice plants to generate a triangular mesh in the *xy*-plane and then computed the corresponding *z* values through comparison with the fitted surface. In this way, they were able to generate a 3D triangle mesh from the fitted surface.

#### Non-uniform rational B-splines

NURBS [236] are mathematical models for generating and representing smooth curves and surfaces in computer graphics. A NURBS surface is completely defined by a list of 3D coordinates of surface control points and associated weights. Fitting techniques of NURBS surfaces are described in Wang et al. [237]. NURBS surfaces can then be triangulated and its surface area approximated by summing the areas of each triangle.

**Fig. 12:**
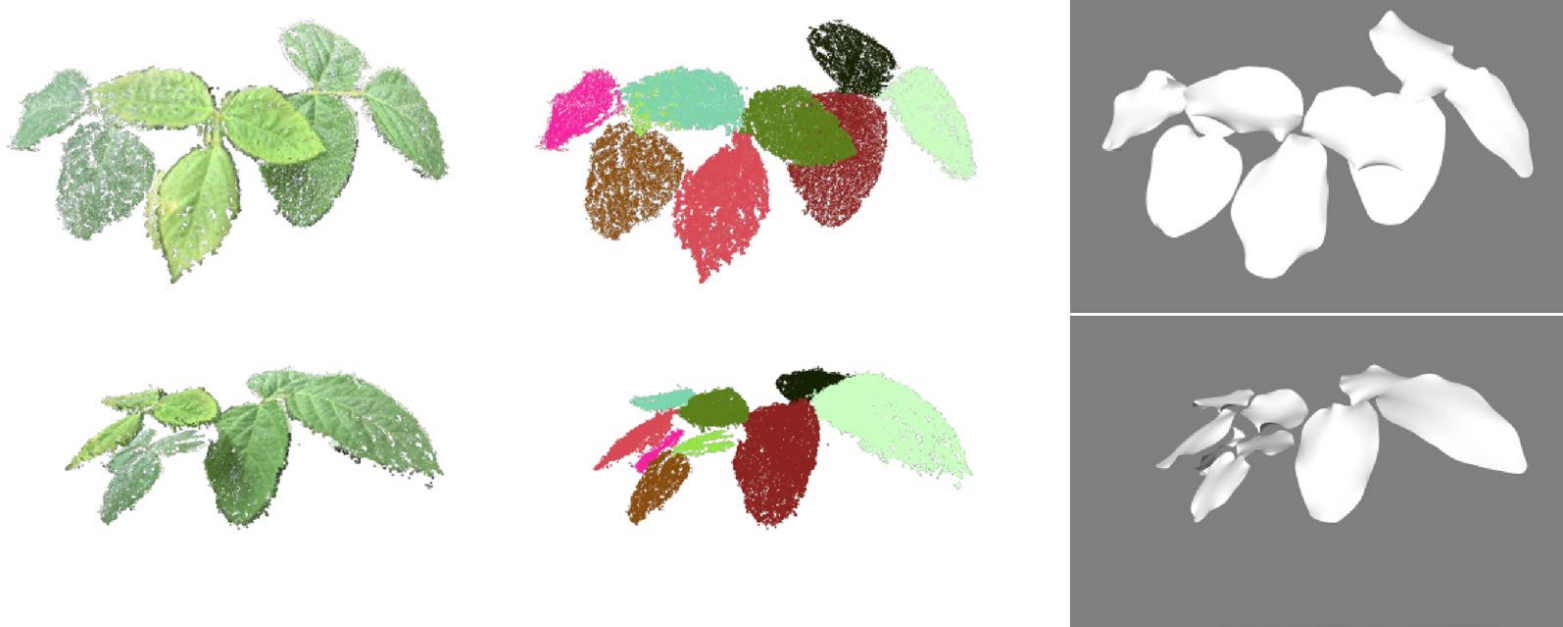
Leaf segmentation and surface fitting using NURBS on a point cloud representation of soybean leaves, by [83]. Reprinted by permission from Springer Nature Customer Service Centre GmbH: Springer Nature, Computer Vision - ECCV 2014 Workshops by Agapito, Bronstein, and Rother, ©2015

NURBS have been applied for the estimation of the surface area of leaves in the following works: Santos et al. [83, 238] first segmented their 3D point clouds of soybean obtained by SfM using spectral clustering, and then fitted NURBS surfaces to the segments corresponding to leaves (Fig. 12); Gélard et al. [239, 240] performed NURBS fitting on segmented leaves of sunflower point clouds obtained by SfM after the stems had been detected and removed using cylinder fitting; and Chaivivatrakul et al. [38] fitted NURBS surfaces to point sets corresponding to maize leaves after these had been mapped onto an underlying surface by MLS.

#### Cylinder fitting

Often stems of plants can be locally represented as a cylinder. A cylinder fitting procedure for oak trees based on least-squares fitting is described in Pfeifer et al. [241]. Paulus et al. [30] applied a similar procedure on stems in 3D laser scanned point clouds of barley. This was done after the segmentation of leaves and stems using PFH. The fitted cylinders allowed them to accurately estimate stem length. Gélard et al. [240] found that cylinder fitting didn’t provide satisfactory results when stems are curved, so they developed an alternative procedure in which they propagated a ring with neighborhood and normal constraints vertically along the stem of a sunflower point cloud to model the stem as a curved tube.

### Trait estimation

After the challenging steps of skeletonization, segmentation and/or surface reconstruction, the measurement of traits on either whole plants, or individual plant organs is often relatively straightforward and many different approaches may yield sufficiently good estimates. Measuring these features is important for a large number of tasks [242], including quantifying plant biomass and yield [243], understanding plant response to stressful conditions [196], mapping genotypes and building predictive structural and functional models of plant growth [244].

#### Whole plant measurements

*Convex hull* The convex hull is defined as the shape of an object which is created by joining its outermost points. The volume of the convex hull of a whole plant can be an indicator for the size of a plant. In root systems, it may be used as an indicator of the extent of soil exploration [245]. Calculating the convex hull of a point cloud requires minimal preprocessing, but provides only a very rough indicator. The convex hull of tomato plant point clouds has been estimated by Rose et al. [85]. The convex hull was estimated on root systems of two *Oryza sativa* (rice) genotypes (Azucena and IR64) by Clark et al. [187], of barley plants by Mairhover et al. [61], and of Rice (Bala $\times$ Azucena) plants by Topp et al. [245].

*Height* Height in point clouds can be simply defined as the maximal distance between points belonging to a plant or root system projected on the vertical axis, such as in Paulus et al. [36] on sugar beet taproots and Nguyen et al. [26] for cabbage and cucumber seedlings. Height can also be easily derived from top-view depth images without much processing as the difference between the ground and the closest pixel in the image, as done by Chéné et al. [110] on rosebushes and Cao et al. [14] on soybean plants.

More robust measures for plant height may be calculated as, for example, by Kjaer and Ottosen [32] where points were arranged in percentiles in relation to their distance from the top-view scanner, and the average of the 80th–90th percentile points was treated as a more robust estimate of rapeseed plant height.

*Area and volume* In the case of point cloud representations, plant area and volume are usually estimated based on 3D meshes. The surface area of a mesh can easily be determined by adding up the area of triangular mesh faces determined by Heron’s formula. The volume of a mesh can be determined by the method described in [246]. Chaudhury et al. [156] calculated total plant surface and volume from an $\alpha$-shape triangulated surface of thale cress plants in this way.

When the plant is represented as a voxel grid or octree, and this representation is precise enough, the volume can be estimated by summing the volumes of all the voxels covering the plant, as was done by Scharr et al. [104] on maize and banana seedlings. However, the authors found that voxel carving methods led to overestimates of volumes due to missed concavities and occlusions.

The surface area of a voxel grid or octree could be estimated by first deriving a meshed surface, which can be obtained with the Marching Cubes algorithm [162].

*Number of leaves* When a segmentation method was able to discriminate between leaves and stems in point clouds or voxel representations, the number of leaves can be derived by counting the number of connected components, after converting the leaf points into a graph in the case of point clouds.

**Fig. 13:**
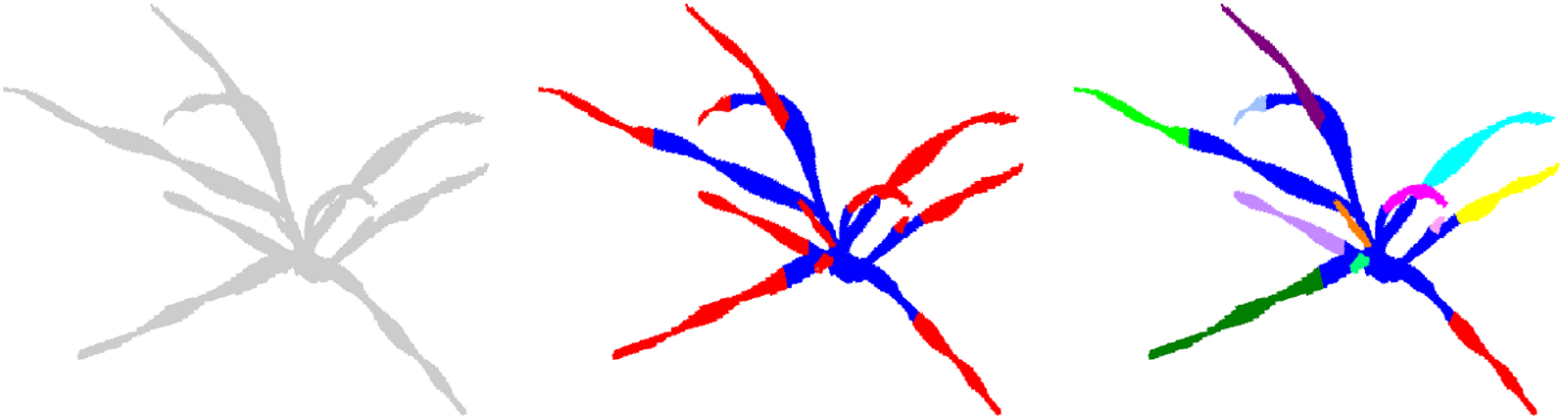
Illustration of the leaf counting method used by [103]. A 3D surface model of barley is segmented based on the eigenvalues of second-moments tensors of the surface, after which connected components corresponding to the distal parts of leaves are counted, to yield the number of leaves of the plant. Reprinted by permission from Springer Nature Customer Service Centre GmbH: Springer Nature, Computer Vision - ECCV 2014 Workshops by Agapito, Bronstein, and Rother, ©2015

In monocot crops leaves are very elongated and not always easily distinguishable from stems. However, an accurate segmentation between leaves and stems is not necessary when the aim is leaf counting. For example, Klodt and Cremers [103] discriminated between only the distal parts of leaves and the rest of barley plants by analyzing gradient directions of the 3D shape (Fig. 13), which was sufficient to count leaves. Another strategy for plants with elongated leaves might be to count leaf tips, which may be represented by the endpoints of a curve skeleton of the plant.

*Petiole length and angle* Cao et al. [14] constructed 3D models of soybean plants based on SfM and measured the petiole length as the length of the longest petiole at the front view and the petiole angle as the angle between a petiole and the stem using the CloudCompare software.

#### Plant organ measurements

*Stem or root dimensions* Stem and internode lengths can be based on curve skeletons or cylinder fits. Paulus et al. [35] derived cumulated stem height from cylinder fits on the stems of barley plants. Golbach et al. [101] used the skeleton of the voxels representing the stem of tomato seedlings.

Using the graph of a skeleton, the lengths of internodes can be estimated by measuring the geodesic distance between branch points using Dijkstra’s algorithm. This was demonstrated by Balfer et al. [247] on a berryless grape cluster which was skeletonized by the method of Livny et al. [177].

**Fig. 14:**
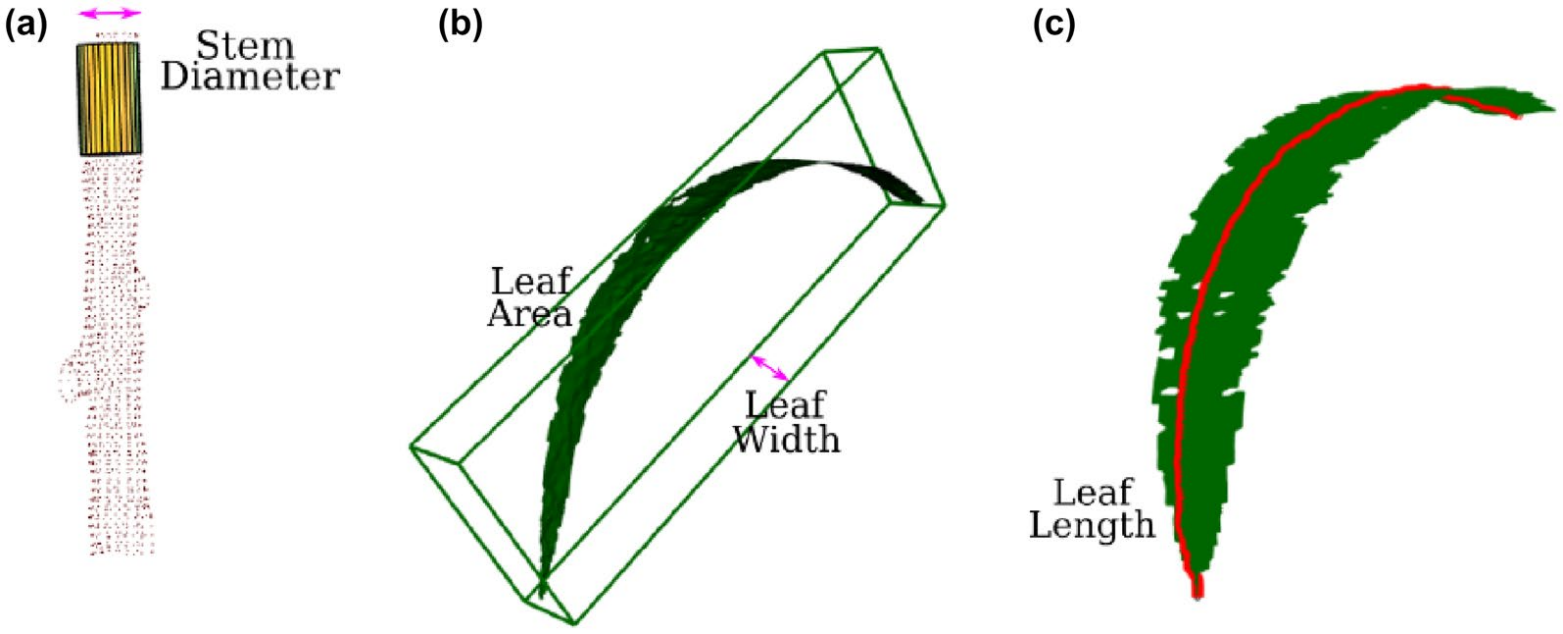
Example of the 3D measurements of plant organs as used by [248]. Stem diameters were estimated by fitting cylinder shapes to stem point cloud segments (**a**), leaf widths by determining the oriented bounding box around leaf point cloud segments and measuring their shortest dimension (**b**), and leaf lengths by computing the shortest paths connecting the furthest points on the leaf surface meshes (**c**). Reprinted with permission from the author

Stem or root widths are often estimated by cylinder fitting. For example, Sodhi et al. [227, 248] fitted primitive cylinder shapes to the segmented stem point cloud of maize plants to extract the stem diameter (Fig. 14).

*Leaf dimensions* Two of the most important architectural traits are leaf angle and leaf area index that have influence on light interception and canopy photosynthesis [130, 249, 250].

The most natural representation for the estimation of leaf dimensions is a mesh surface. Leaf area is then easily estimated as the sum of the area of triangular mesh faces as was done by Sodhi et al. for sorghum [227], by Gélard et al. for sunflowers [239, 240] and by Chaivivatrakul et al. for maize [38]. Leaf base point is defined as the closest point to the stem point clouds [130]. Leaf length and width can be calculated by determining the longest geodesic shortest path on the mesh expressed as a graph, by applying Dijkstra’s algorithm [171]. Liu et al. [130] implemented a three-step procedure to find the leaf tip point of maize plants and then defined the leaf length as the distance of the shortest path between the leaf base and the leaf tip. Sodhi et al. [227, 248] estimated leaf width of sorghum plants by determining an oriented bounding box around a leaf point set, whose sides are directed towards the principal axes of the point set. The leaf width is then the second longest dimension of the bounding box (Fig. 14).

Golbach et al. [101] instead derived the leaf dimensions of tomato plant seedlings directly from a voxel representation to minimize computing time. After segmentation they determined leaf length as the distance between the two points on the surface of the leaf which are furthest away from each other. To correct for the curved shape of the leaves, they added an additional point on the leaf surface halfway between these points. For the leaf width, they searched for the maximum leaf width perpendicular to the three point leaf midrib which was used for the leaf length. For leaf area they used an approximation based on the number of surface voxels. The authors choose rather crude measurements and may have sacrificed some precision in favour of speed.

Duan et al. [169] based their measurements of leaf lengths and widths of wheat seedlings on polynomial regression fits through segmented leaf point clouds. They identified leaf edges according to the 90th percentile on either side of the leaf midrib using quantile regression, to account for the presence of noise.

*Ear or fruit volumes* Plant yields may be approximated by the estimated volumes of plant ears or fruits. For example, after segmentation based on PFH, Paulus et al. [35] found that ear weight, kernel weight and number of kernels in wheat plants was correlated with their estimates of ear volume, which they obtained by estimating $\alpha$-shape volumes on the point sets corresponding to the ears.

#### Canopy level measurements

When 3D acquisition methods don’t provide sufficient detail to allow for measurement of individual plant organs, such as when applied on larger scales in the field, useful information can still be extracted on the level of crop or tree canopies. Examples of such traits are canopy surface height, vertical plant area density distribution, leaf area index, or leaf angle distribution.

Cao et al. [14] measured the canopy width of soybean plants as the maximum plant canopy width from the projection on the front view of 3D points clouds.

*Canopy profiling* LiDAR has a certain capacity to penetrate canopies, so that in LiDAR the frequency of laser interception by a canopy can be used as an index of foliage area at each height. This canopy profiling by airborne LiDAR has been deployed mostly in the context of ecological studies on forest stands [251, 252]. However, Hosoi and Omasa [253] used a high-resolution portable scanning LiDAR together with a mirror for vertical plant area density profiling of a rice canopy at different growth stages. Their method for the estimation of leaf area density is based on a voxel model, and is described in [254]. The leaf area index can then be derived from the vertical integration of leaf area density values.

Cabrera et al. [255] instead used 3D voxel grid representations of individual maize plants to study light interception of maize plant communities, by creating virtual canopies of maize. In the virtual canopy, the cumulative leaf area and the average leaf angles were determined based on the 3D representations of individual plants. These measures were combined with a model of incident light in the greenhouse, so that the local light interception by the canopy could be estimated.

*Leaf angle distribution* 3D image acquisition methods provide the opportunity to study temporal patterns in the orientation of leaves, which is a highly dynamic trait that changes in response to fluctuations in the environment. Biskup et al. [77] presented a method based on top-view stereo imaging. Their depth images were subjected to a graph-based segmentation algorithm [256] to obtain a rough segmentation of individual leaves of soybean plants, after which planes were fitted to each segment using RANSAC to determine leaf inclination angles. Müller-Linow et al. [108] presented a software tool to analyze leaf angles in crop canopies based on the same set of methods.

## Machine learning techniques for plant phenotyping

Machine Learning (ML) is the scientific study of algorithms and statistical models used by a computer system to perform a specific task without explicit instructions, but relying only on patterns and inference. With sensors and acquisition systems for plant phenotyping widely available and used to generate large amounts of imaging data, the main challenge lies in translating the high-dimensional raw imaging data into the quantification of relevant plant traits. In the past, this was done through manually engineered image processing methods, as discussed in the previous sections, but to deal with the difficulties of complex plants, non-controlled, or cluttered environments, ML is gaining in popularity. Classical approaches in computer vision consist in general of two major steps, feature extraction using those manually engineered image processing methods and decision making using ML methods, while modern Deep Learning (DL) approaches take an integrated, end-to-end approach, in which features are learned at the same time as the inference is performed. Moreover, DL models are often more complex than classical ML models, resulting in much greater discriminative and predictive power [257], with spectacular results in different application areas [258, 259].

Machine learning for plant phenotyping, and deep learning in particular, is an actively developing field. To the best of our knowledge, most of the ML methods have been used in plant segmentation, though ML is starting to find applications outside of plant segmentation as well, for example in denoising or registering the plant point cloud [34, 186]. Indeed, we believe that ML is expected to impact *all* aspects of plant phenotyping, leading to significant improvements in the current state-of-the-art in the coming years. For example, new DL architectures could be developed and adopted for 3D and multi-modal data processing like skeleton extraction, branch-pattern classification and plant-development understanding [260]. Furthermore, ML algorithms can be used to analyse the data from high-throughput phenotyping experiments, and may alleviate the problem of missing data, leading to the identification of new correlations and plant traits that were previously difficult to detect.

**Fig. 15:**
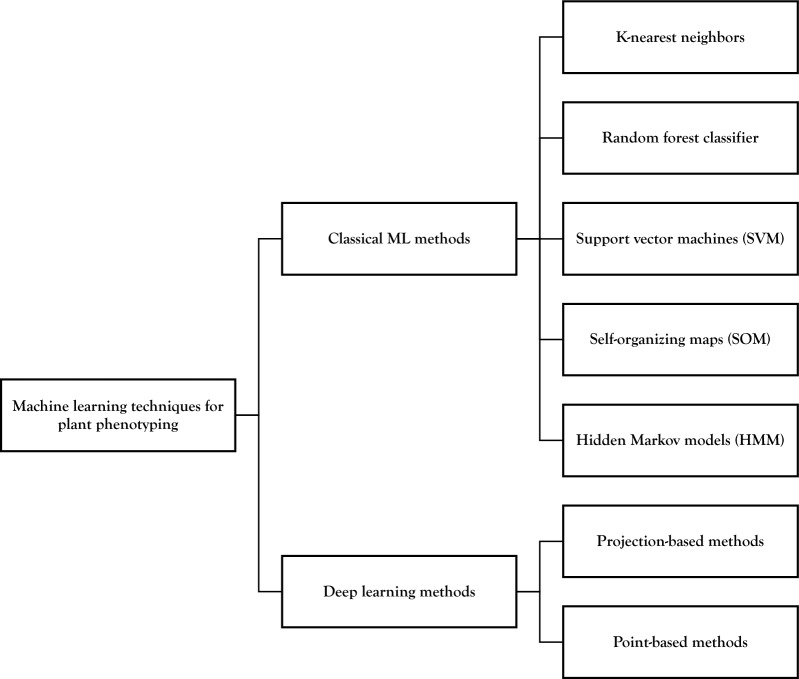
Overview of ML techniques for 3D plant phenotyping

A full list of papers and plants using these techniques can be found in Table 3, under the header “Machine Learning Techniques”. Moreover, an overview of the topics covered in this section is presented in Fig. 15.

### Classical ML methods

In this section, we review some classical machine learning algorithms that are used for plant segmentation. Compared to DL methods, these techniques can often be used efficiently on relatively small datasets, and have a less complex structure, but they are usually less accurate [261].

#### K-nearest neighbors

The KNN algorithm is an ML classifier which uses the concept of proximity to make predictions about the grouping of individual data points, working off the assumption that similar points can be found near one another. The KNN algorithm can also be used for clustering, with applications for denoising and downsampling in plant phenotyping.

Wu et al. [186] proposed a clustering algorithm based on an implementation of the KNN algorithm by Connor and Kumar [262] to denoise point cloud data for maize plants. Along similar lines, Chebrolu et al. [34] and Magistri et al. [161] used KNN clustering to refine the initial segmentation of tomato and maize plants by discarding small clusters and assigning each discarded point to one of the remaining clusters. Gibbs et al. [81] implemented an efficient KNN algorithm for the downsampling of plant shoot point clouds, and applied their method to different plants (bromeliad species, aloe vera, cordyline species, Brassica sp., chili, and pumpkin).

#### Random forest classifier

The random forest classifier (RFC), first proposed by Breiman [263], is an ensemble learning method in which a multitude of decision trees are constructed during training time, and predictions from the individual trees are pooled for inference.

Straub et al. [135] used two applications of the RFC algorithm to build a tree model for meadow orchard trees. First, the point cloud is separated into two classes, “ground” and “tree”, and secondly the “tree” class is further processed to filter out noise caused by the fine structure of the tree branches, which were photographed against the sky and differed strongly in their color values from the real branch points.

Dutagaci et al. [264] used a volumetric approach, where an RFC was trained on local features derived from the eigenvalues of the local covariance matrix (intuitively speaking, these local features serve to discriminate leaf and stem points by distinguishing flat structures from elongated, thin structures). They applied their method on rosebush plants, and showed that this voxel classification method through local features gave the best overall performance for leaf and stem classification among four baseline methods they had defined.

#### Support vector machines

Support vector machines (SVMs) are a commonly used choice for binary classification problems and can perform nonlinear classification through the use of kernels.

Sodhi et al. [227] used an SVM classifier to classify each point of a 3D point cloud of maize plants as either belonging to the stem or to a leaf. Chebrolu et al. [34] and Magistri et al. [161] used a standard SVM classifier with FPFH features to perform a segmentation step aiming at grouping together points belonging to the same plant organ, a single leaf instance, or the stem.

Zhou et al. [84] evaluated the performance of two SVMs (with different polynomial kernels) and two other machine learning methods (boosting and k-means clustering) for the segmentation of soybean plants at early growth stages using 3D point cloud data built from 2D images. They found that the SVM with a linear kernel (applied to histogram of oriented gradients (HOG) features) outperformed the SVM with a 2nd-order polynomial kernel in distinguishing between plant features and background. In case of overlapping plants separation, they showed that the SVM with a linear kernel had the smallest error rate, while for background removal and non-overlapping plants separation, *k*-means clustering performed best. They also showed that *k*-means clustering outperformed two other methods (the SVM with linear kernel and boosting) in the aspect of processing efficiency and segmentation accuracy.

#### Self-organizing maps

Self-organizing maps (SOMs) are unsupervised neural networks developed by Kohonen [265] using the concept of competitive learning instead of back-propagation [34]. SOMs map multi-dimensional data onto lower-dimensional subspaces where geometric relationships between points indicate their similarity.

Chebrolu et al. [34] and Magistri et al. [161] assigned each point in the point cloud to a plant organ (stem or leaf) and then applied SOMs to learn the nodes of the skeleton structure for each plant organ, after which these nodes were used to build the plant skeleton structure of maize and tomato plants.

#### Hidden Markov models

Hidden Markov models (HMMs) are probabilistic models in which an unobservable (“hidden”) Markov process influences an observable process [266]. HMMs have been used in plant phenotyping to determine correspondences between time-series data of tomato and maize plants by Chebrolu et al. [34] (cf. "3D point set registration" section). Because of their probabilistic nature, HMMs are well suited for cases where the observed measurements suffer from noise and other imperfections.

### Deep learning methods

Image segmentation can be categorized into semantic segmentation and instance segmentation. The goal of semantic image segmentation is to label each pixel of an image with a corresponding class of what is being represented. Instance segmentation is considered the next step after semantic segmentation and its main purpose is to represent objects of the same class split into different instances.

Many DL approaches have been developed for the segmentation of 2D images [267–274]. However, most DL methods for segmentation are a priori only applicable to images defined on a regular grid-like structure (so that, for example, convolutions can be applied for feature extraction [160]) and are not well-suited for unstructured data such as 3D point clouds or models [268, 275–277].

Moreover, the problem of performing semantic segmentation directly on 3D data is challenging due to the limited availability of 3D datasets with segmentation annotations. Semantic segmentation techniques for 3D point clouds are further divided into two groups: projection-based methods and point-based methods [277], which are discussed below.

#### Projection-based methods

Projection-based techniques first project the 3D point cloud onto an intermediary 2D representation that can be segmented using 2D networks, and then construct a segmentation for the full 3D point cloud out of these intermediary segmentation results. The advantage is that established 2D segmentation networks can be used, but due to the intermediate representation, some loss of spatial and geometrical information is inevitable [277–280].

According to the type of intermediary representation, several categories of projection-based methods can be distinguished; in this paper we discuss the multi-view, volumetric, and lattice representation. Another representation, the spherical representation (see, e.g., [281]) retains more geometrical and spatial information than for example the multi-view representation, but as it currently has no applications in plant phenotyping as far as we know, it is not discussed in this paper.

*Multi-view representation* These methods project the 3D shape or point cloud onto multiple 2D images or views, and then extract feature from the 2D data by using existing models. Two of most popular networks in this category are MVCNN [282] which analyses the data from multiple perspectives using convolutional neural networks (CNN), and SnapNet [283], which uses snapshots of the point cloud to generate RGB and depth images to work around the problem of information loss.

Determining the number of projections to use, the viewing angle for each projection, and the way to re-project the segmented models from 2D to 3D space, are the main difficulties associated with this class of techniques [276, 284].

Shi et al. [2] applied a multi-view approach and used a slightly modified version of VGG-16 [285], a fully convolutional network (FCN [286]), for semantic segmentation, and a Mask Recurrent Convolutional Neural Network (R-CNN [287]) for instance segmentation on 2D images of tomato seedling plants and then combined the 2D segmentation results in a 3D point cloud. They applied this segmentation method on 2D data as well and showed that this multi-view 3D approach outperforms the 2D approach both for semantic and instance segmentation.

*Volumetric representation* These methods transform the unstructured 3D point cloud into a regular spatial grid (voxelisation), and then train a neural network on this grid to perform the segmentation. Some popular architectures in this group, which are currently not yet used for plant phenotyping, are VoxNet [288], OctNet [289], and SEGCloud [290]. Volumetric techniques produce reasonable results on small point clouds, but are memory-intensive and hence may struggle on complex datasets.

Dutagaci et al. [264] compared segmentation results for rosebush plants obtained using the 3D U-Net [291] architecture with three other methods for segmentation, namely Local Features on Volumetric Data (LFVD) and a supervised and unsupervised version of Local Features on Point Clouds (LFPC). They found that the 3D U-Net gave the lowest performance whereas the combination of the LFVD feature extraction method with an RFC obtained the best performance for segmentation.

*Lattice representation* This representation converts a point cloud into sparse, discrete elements (lattices). The sparsity of the extracted features is adjustable and these methods typically have lower memory and computational requirements than simple voxelisation. SPLATNet [292], LatticeNet [293], and MinkowskiNet [294] fall in this category.

Schunck et al. [160] used three different DL architectures for the semantic segmentation of the raw point cloud into leaf, stem and ground: PointNet, PointNet++, and LatticeNet [293, 295]. LatticeNet applies convolutions on a permutohedral lattice while the PointNet-based methods (See "Point-based methods" section) rely on pooling point features to obtain their internal representation. The authors trained these networks for tomato and maize separately, using 5 plants for training and 2 plants for testing. All three methods achieved high intersection over union (IoU) in the leaf and ground class. The PointNet-based methods struggled with the stem class because it contained relatively few points while LatticeNet achieved good results for all classes.

#### Point-based methods

Point-based methods work directly on point clouds without introducing any intermediate representation. Hence, they are able to use the full set of raw point cloud data, with all of its geometrical and spatial features. These methods are widely used and the subject of active development, and can be roughly divided into five categories: pointwise methods, convolution methods, recurrent neural network (RNN)-based methods, recursive neural network (RvNN)-based methods, and graph-based methods.

Graph-based methods make use of the graph structure of the point cloud, often applying a DGCNN network [296, 297] as the underlying architecture. Since graph-based methods have to the best of our knowledge no applications in plant phenotyping at the moment, they are not discussed in this paper.

*Pointwise methods* PointNet, introduced by Qi et al. [298], is a pioneering effort in this regard and provides a unified approach to a number of 3D recognition tasks including object classification and segmentation. However, this method has trouble capturing local structures, limiting its ability to recognize fine-grained patterns and to generalize to complex scenes.

Li et al. [299] built an automated organ-level point cloud segmentation system for maize plants, using Label3DMaize [203] to label data from a high-throughput data acquisition platform for individual plants, and PointNet to implement stem-leaf and organ instance segmentation.

Later, Qi et al. [300] introduced PointNet++ which is a hierarchical neural network that applies PointNet recursively on a nested partitioning of the input point set. While PointNet used a single max-pooling operation to aggregate the entire point set, their new architecture builds a hierarchical grouping of points into progressively larger and larger local regions along the hierarchy.

Heiwolt et al. [301] applied the PointNet++ architecture, adjusted for point-wise segmentation applications, on tomato plants and showed that this network was able to successfully predict per-point semantic annotations for soil, leaves, and stems directly from point cloud data.

To better incorporate local geometric structures, the last years have seen a number of improvements upon the Pointnet architecture, including PointSIFT [302], SGPN [303], DGCNN [296], LDGCNN [304], SRN-PointNet++ [305], ASIS [306], PointGCR [307], and PointNGCNN [308]. To the best of our knowledge, these improved methods have yet to be applied to plant phenotyping.

*Convolution methods* As point clouds consist of irregularly spaced, unordered points, convolution operators designed for regular, grid-based data cannot be applied directly.

To address this issue, Li et al. [309] introduced PointCNN which generalizes the design of a CNN to be applicable to point clouds. Ao et al. [310] applied PointCNN on morphological characteristics of the maize plant to segment stem and leaves of the individual maize plants in field environments. They showed that their approach overcomes the major challenges in organ-level phenotypic trait extraction associated with the organ segmentation.

Wu et al. [275] proposed PointConv, extending traditional image convolution to 3D point cloud data with non-uniform sampling. They found that PointConv outperforms networks like PointNet and PointNet++ on several widely used datasets in terms of accuracy and IoU.

Gong et al. [311] developed Panicle-3D, which has higher segmentation accuracy and faster network convergence speed than PointConv, and applied the proposed network on point clouds from rice panicles. A drawback of the method is that it requires large volumes of labelled data to train the network.

Chen et al. [312] developed the DeeplabV3+ network for semantic segmentation, using the convolutional neural network (CNN) structure of the DeeplabV3 network [272] as a starting point and adding a decoder module for refining the segmentation results, especially along object boundaries. Chen et al. [90] used this network to segment banana central stocks.

As an alternative convolution method, we also mention the work of Jin et al. [313], who proposed a voxel-based CNN (VCNN) to do semantic segmentation and leaf instance segmentation on the collected LiDAR point clouds of 3000 maize plants.

Despite these ongoing efforts, three main challenges still exist: (a) the lack of well-labelled 3D plant datasets, (b) achieving highly accurate point-level organ semantic and instance segmentation, and (c) the generalization of the proposed method to other plant species (since most DL approaches are currently focused on a single species at a time).

To address the third challenge, Li et al. [276] proposed a dual-function point cloud segmentation network named PlantNet, the first architecture to be able to work on several plant species, and applied their method on tobacco, tomato, and sorghum plants. They also provided a well-labelled point cloud dataset for plant stem-leaf semantic segmentation and leaf instance segmentation containing 5460 LiDAR-scanned crops (including 1050 labelled tobacco plants, 3120 tomato plants, and 1290 sorghum plants).

*RNN-based methods* These techniques have recently been used for segmentation because they are able to capture inherent context features and enhance the connection between local features of the point cloud. They first transform a block of points into multi-scale blocks or grid blocks, after which features are extracted by using PointNet. These features are then fed into recurrent consolidation units to obtain the output-level context. One of the most popular networks in this category is 3DCNN-DQN-RNN [314].

Bernotas et al. [17] used two different neural network architectures, an RNN and an R-CNN. The R-CNN was pre-trained using transfer learning weights generated on the Common Objects in Context (COCO) data set and both networks were trained starting with random initial weights. Comparing both approaches on thale cress rosettes, the most accurate leaf segmentation results were achieved with models based on the R-CNN architecture using pre-trained weights.

*RvNN-based methods* These networks, developed by Socher et al. [315], can achieve predictions in a hierarchical structure. In this category, PartNet, presented by Yu et al. [316], is a DL model for top-down hierarchical, fine-grained segmentation of 3D shapes. This network takes a 3D point cloud as input and then performs a top-down decomposition and outputs a segmented point cloud at the level of part instances.

Wang et al. [44] applied PartNet for instance segmentation on their 3D plant dataset of lettuce consisting of a mixture of real and synthetic data. They showed that the constructed PartNet network had the potential to accurately segment the 3D point cloud leaf instances of lettuce.

## Perspectives

As this paper has shown, there exists an abundance of automated solutions for 3D phenotyping. It remains a challenge, however, to find a low-cost, high-throughput 3D reconstruction method that can handle different types of plants and plant traits, especially considering difficulties such as occlusion. All 3D measuring methods have in common that with increasing plant age, the complexity and thus the amount of occlusion increases. Even though this problem can be addressed in part by using more viewpoints, occlusion will always be present, independent of the type of sensor, the number of viewpoints or the sensor setup, as the inner center of the plant will at a specific moment in time be occluded by the plant (leaves) itself. Although some solutions exist that use volumetry, such as using MRI or radar systems, a more complex and expensive measuring setup should be taken into account [18]. Furthermore, many methods and solutions can be applied on individual plants but not on dense canopies. SfM, for example, obtains good results for the 3D reconstruction of plants (and is additionally one of the most cost-effective methods), but it is not suitable for very dense canopies [317].

Performing a reconstruction of real scenes in 3D phenotyping as a function of time is a challenging but important task, since it will allow for dynamic traits to be considered, such as growth rates which could provide information about the growth behavior of plants throughout their different growth stages. The detection of such variations in growth rates might permit the identification of genes controlling plant growth patterns or the selection of plant genotypes with strong resistance for high production or harvesting strategies [43].

Registering plants over the course of time is challenging due to the anisotropic growth, changing topology, and non-rigid motion in between the time of measurements. For the registration problem, correspondences between point clouds of plants, taken at different points in time, should be determined and then should be registered using a non-rigid registration approach. Regarding our previous discussion about registration (see "3D point set registration" section), point cloud registration for non-rigid plants is itself a challenging problem especially when some correspondences are missed and still is an open area of research. Focusing on detecting key correspondences can be considered as a solution to overcome this problem.

One area in which much progress can be foreseen for 3D phenotyping, and especially for the task of segmenting 3D representations of plants, is the application of machine learning algorithms (see "Machine learning techniques for plant phenotyping" section). As discussed, most of the ML methods have been used in plant segmentation, and finding applications outside of plant segmentation or adapting these ML methods to cover different areas in the plant domain can be an area of research in the future, for example in denoising or registering the plant point cloud [34, 186].

Deep learning presents many opportunities for image-based plant phenotyping, but these techniques typically require large and diverse amounts of ground-truthed training data to learn generalizable models without providing a priori an engineered algorithm for performing the task. In most vision-based tasks where deep learning shows a significant advantage over engineered methods, such as image segmentation, classification, and detection and localization of specific objects in a scene, the size of the dataset is typically in the order of tens of thousands to tens of millions of images. This requirement is challenging, however, for applications in the plant phenotyping field, where available datasets are often small and the costs associated with generating new data are high [1]. Furthermore, the manual segmentation of plant images is a cumbersome, time-consuming, and error-prone process. To alleviate this problem, Ubbens et al. [1] proposed a new method for augmenting plant phenotyping datasets using rendered images of synthetic plants, while Chaudhury et al. [318] proposed a generalized approach to generate annotated 3D point cloud data of a thale cress plant using some artificial plant models.

So far several comprehensive collections of benchmark datasets for plant phenotyping with annotations have been made publicly available: the dataset of Khanna et al. [319] containing biweekly color images, infra-red stereo image pairs, and hyperspectral camera images of sugar beet plants along with applied treatment and weather conditions of the surroundings, collected over two months; the ROSE-X dataset of Dutagaci et al. [264] including 11 fully annotated 3D models of real rosebush plants obtained through X-Ray imaging; the Pheno4D dataset of Schunck et al. [160] containing highly accurate and registered point clouds of 7 maize and 7 tomato plants collected on different days (approximately 260 million 3D points); the multi-modality dataset MSU-PID of Cruz et al. [320] containing segmented top-view RGB images of growing thale cress and bean plants; the CVPPP leaf segmentation dataset of Minervini et al. [196] containing segmented top-view images of growing thale cress and tobacco plants; the KOMATSUNA dataset of Uchiyama et al. [195] containing segmented top-view RGB images of spinach (Komatsuna) plants; and the Annotated Crop Image Database of Pound et al. [257] containing images and annotations of wheat spikes and spikelets. Among them, the three datasets of MSU-PID, CVPPP, and KOMATSUNA consist of raw and annotated 2D color images of rosette plants taken from above. The analysis of these images involves segmenting individual and overlapping leaves, for which neural networks have had the greatest success [321–326].

As more benchmark datasets for 2D and 3D plant phenotyping are being made available, the application of neural networks is expected to achieve a similar level of success as in other areas.

Fully automated 3D segmentation approaches for plant point cloud which could cope with a wide range of different shaped plants are a challenging problem, and also are a bottleneck in achieving big data processing of 3D plant phenotyping [299]. Recently, Wei et al. [327] presented a novel point cloud segmentation network called BushNet which is for the semantic segmentation of bush point clouds in large-scale environments. However, there is no application on plant cases so far.

In this regard, future research trends can focus on the adaptation and customization of newly developed ML models for applications in plant phenotyping, and also on generalizing capabilities of current models to be used on different kinds of plants. Segmentation is not the only part of the 3D plant phenotyping which can get the benefit of DL methods. However, DL is currently not frequently used for other phenotyping steps such as skeletonization and denoising. This, too, could form a fruitful area for future research, to assist e.g. with alleviating the impact of noise and missing data.

Last, we foresee that AI-assisted plant phenotyping may have the potential to optimize pest control and improve crop yield, through the large-scale analysis of plant traits and the identification of signs of biotic and abiotic stresses, such as pest damage, drought, and high temperatures. This is especially the case as ML methods have enabled practitioners to move beyond single-plant phenotyping to estimate plant traits at the canopy or field level, providing a more comprehensive understanding of how stressors impact overall crop health, thus improving agricultural productivity and sustainability.

## Conclusion

This review provides a broad but non-exhaustive overview of processing and analysis methods applied or applicable in 3D plant phenotyping. As shown, the set of techniques applicable in this field is very diverse, which contributes to the complexity of the task of 3D plant phenotyping. As this is an expanding field, we foresee that additional methods not mentioned in this review will be explored in the future.

## Data Availability

Not applicable.

## Abbreviations

μCT: Micro Computed Tomography
2D: Two-dimensional
3D: Three-dimensional
CNN: Convolutional Neural Network
COCO: Common Objects in Context
CPD: Coherent Point Drift
CRF: Conditional Random Field
CT: Computed tomography
DBSCAN: Density-based spatial clustering of applications with noise
DL: Deep learning
ERT: Electrical Resistance Tomography
FCN: Fully convolutional network
FPFH: Fast Point Feature Histogram
GMM: Gaussian Mixture Model
HMM: Hidden Markov Model
HOG: Histogram of oriented gradients
HSV: Hue, saturation and value
ICP: Iterative Closest Point
IoU: Intersection over union
KNN: K-nearest neighbors
LFPC: Local Features on Point Cloud
LFVD: Local feature on volumetric data
LiDAR: Light detection and ranging
LOESS: Locally Estimated Scatterplot Smoothing
ML: Machine learning
MLS: Moving Least Squares
MOBB: Minimum oriented bounding box
MRI: Magnetic Resonance Imaging
MSAC: M-estimator Sample Consensus
MVS: Multi-view stereo
NT: Neutron tomography
NURBS: Non-uniform rational basis splines
PCA: Principal Component Analysis
PFH: Point Feature Histogram
PMVS: Patch-based Multi-View Stereo
PS: Photometric Stereo
R-CNN: R-convolutional Neural Network
RAIN: Randomly Intercepted Nodes
RANSAC: Random Sample Consensus
RBOF: Radius-based outlier filter
RFC: Random Forest Classifier
RNN: Recurrent neural network
RvNN: Recursive neural network
SD: Standard deviation
SfM: Structure from Motion
SFS: Shape-from-silhouette
SOM: Self-organizing maps
SOR: Statistical outlier removal
SVM: Support vector machine
TLS: Terrestrial laser scanner
ToF: Time of Flight
